# Characterization of [^3^H]Propionylated Human Peptide YY-A New Probe for Neuropeptide Y Y_2_ Receptor Binding Studies

**DOI:** 10.1021/acsptsci.4c00666

**Published:** 2025-02-25

**Authors:** Franziska Schettler, Albert O. Gattor, Pierre Koch, Max Keller

**Affiliations:** Institute of Pharmacy, Faculty of Chemistry and Pharmacy, University of Regensburg, Universitätsstraße 31, Regensburg D-93040, Germany

**Keywords:** neuropeptide Y, peptide YY, Y_2_ receptor, radioligand, binding assay

## Abstract

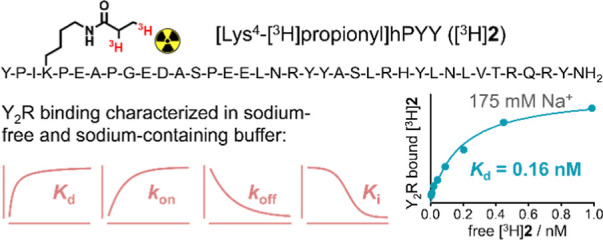

The neuropeptide Y (NPY) Y_2_ receptor (Y_2_R) is a G-protein-coupled receptor that is involved in the regulation of various physiological processes such as neurotransmitter release, bone metabolism, and memory. Consequently, the Y_2_R represents a potential drug target, e.g., for the treatment of epilepsy and mood disorders. Until now, the determination of the Y_2_R binding affinities of Y_2_R ligands has primarily been performed using ^125^I-labeled derivatives of the endogenous Y_2_R agonists NPY and peptide YY (PYY). A tritium-labeled NPY derivative has also been used; however, its suitability for binding assays in sodium-containing buffer is doubtful. We synthesized a tritium-labeled PYY derivative by [^3^H]propionylation at Lys^4^ ([^3^H]**2**). The radioligand was characterized by saturation binding, association, and dissociation kinetics and was applied in competition binding assays. Specific binding of [^3^H]**2** at intact Chinese hamster ovary cells expressing the hY_2_R was saturable in both sodium-free buffer (apparent *K*_d_ = 0.016–0.067 nM) and sodium-containing buffer (175 mM Na^+^, apparent *K*_d_ = 0.16–0.18 nM). Competition binding experiments with Y_2_R reference ligands yielded *K*_i_ values, which are in good agreement with the reported Y_2_R binding affinities, showing that [^3^H]**2** represents a useful tritiated tool compound for the determination of Y_2_R binding affinities also in buffers containing sodium at physiological concentrations.

The family of human neuropeptide Y (NPY) receptors comprises four functionally active subtypes, namely the Y_1_R, Y_2_R, Y_4_R, and Y_5_R, which all belong to class A of G-protein-coupled receptors. Activation of all four receptor subtypes is induced by the homologous neuropeptides NPY, peptide YY (PYY), and pancreatic polypeptide (PP).^1^ These endogenous peptides exhibit different Y receptor (YR) affinity and potency profiles. While NPY and PYY exhibit comparable YR affinity profiles (Y_1_R ≈ Y_2_R ≈ Y_5_R > Y_4_R), the binding profile of PP is characterized by a clear preference for Y_4_R over the other YRs. Accordingly, NPY and PYY bind to the Y_2_R with a comparable affinity.^2−4^ Through its wide distribution within the brain,^5−7^ Y_2_R is implicated in numerous physiological processes like food intake,^8,9^ bone formation,^10,11^ or learning and memory.^12,13^ Mainly acting as a presynaptic autoreceptor,^14,15^ Y_2_Rs regulate the release of different neurotransmitters like γ-aminobutyric acid (GABA)^16^ or glutamate.^17,18^ Therefore, the Y_2_R is considered a potential target for the treatment of epilepsy^19^ or mood disorders.^20,21^ To investigate Y_2_R binding of potential drug candidates, radiochemical binding assays using iodine-125 labeled derivatives of NPY ([^125^I]NPY^22,23^) or PYY ([^125^I]pPYY,^24^ [^125^I]PYY(3–36)^25,26^) are commonly applied. Considering the species of the peptidic agonists, human or porcine NPY and human or porcine PYY (sequences shown in Figure 1) are usually used as precursors for radiolabeling.

**Figure 1:**
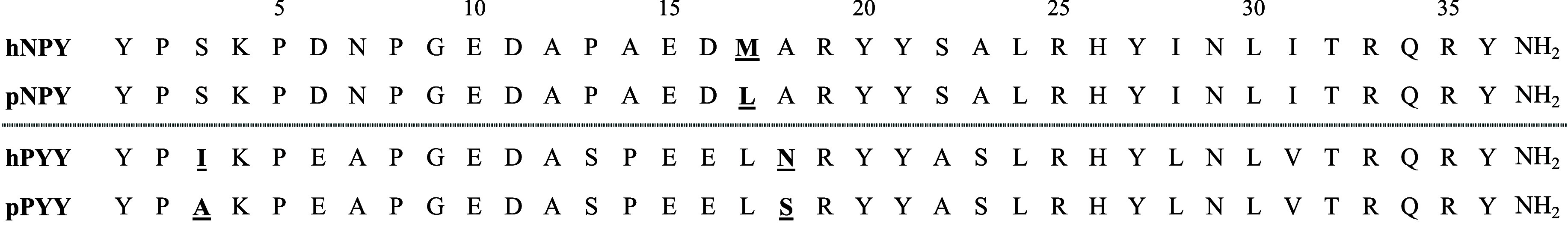
Amino acid sequences of hNPY, pNPY, hPYY, and pPYY. Differences in the amino acid sequence of hNPY and pNPY and differences in the sequence of hPYY and pPYY are indicated bold and underlined.

However, it should be noted that the species of the peptides is often not specified in the respective articles. In the case of NPY, porcine NPY, whose sequence differs from that of human NPY only in position 17 (pNPY: Leu^17^, hNPY: Met^17^, cf. Figure 1), is generally preferred over hNPY for the preparation of labeled NPY derivatives since methionine residues are prone to oxidation even in vitro.^27,28^ Therefore, it is likely that porcine NPY was used if no species is provided for the precursor and the radiolabeled NPY. The reported dissociation constants *K*_d_ (Y_2_R) and the binding assay buffers used for the reported iodine-125 labeled NPY and PYY derivatives are summarized in Table 1.

**Table 1:** Reported Peptidic Radioligands Used for Y_2_R Binding Studies

radioligand	reference	species	*K*_d_ [nM]	cells/tissue	buffer	Na^+^ conc. [mM]
[^125^I]PYY (3–36)	Dumont et al.^25^	not indicated	0.013	rat hippocampal membrane preparation (autoradiography)	KRP	145
	Dumont et al.^36^	not indicated	not reported	rat brain (autoradiography)	KRP	143
	Dumont et al.^37^	not indicated	0.25	HEK293 rY_2_R	KRP	145
[^125^I]PYY	Brothers et al.^38^	not indicated	not reported	HEK293-CNG Y_2_R	DMEM/10 mM HEPES	157^a^
	Shoblock et al.,^39^ Bonaventure et al.^40^	not indicated	not reported	KAN-T hY_2_R	20 mM HEPES	120
	Rose et al.^2^	not indicated	0.27	COS-7 pY_2_R	50 mM HEPES	0
	Gerald et al.^24^	porcine	0.067	cloned human hippocampal Y_2_R	20 mM HEPES-NaOH	30
[^125^I]NPY	Doods et al.^23^	human	not reported	SMS-KAN hY_2_R	MEM/25 mM HEPES	144
	Rose et al.^2^	not indicated	0.58	COS-7 pY_2_R	50 mM HEPES	0
	Grandt et al.^41^	human	not reported	Y_2_-like receptors on CHP 234	10 mM HEPES	175
[Lys^4^-[^3^H]propionyl]pNPY	Ziemek et al.^42^	porcine	0.7	CHO-hY_2_-Gq_i5_-mtAEQ	25 mM HEPES	0
	Konieczny et al.^43^	porcine	0.14	CHO-hY_2_R	25 mM HEPES	0
*N*-[^3^H]propionyl-NPY^b^	Czerwiec et al.^34^	not indicated	0.7	calf hippocampus membrane preparation	Krebs–Ringer/20 mM HEPES	137

a In the respective reference, the origin/manufacturer of the used DMEM is not provided. Given is the sodium concentration, which is usually contained in DMEM (e.g., Thermo Fisher Scientific). KRP, Krebs–Ringer phosphate buffer; DMEM, dulbecco’s modified eagle medium; HEPES, 4-(2-hydroxyethyl)-1-piperazineethanesulfonic acid; MEM, minimum essenial medium.

b Although the name “*N*-[^3^H]propionyl-NPY” rather indicates an N-terminally [^3^H]propionylated NPY derivative, the authors most likely used an NPY derivative, which was [^3^H]propionylated at the ε-amino group of Lys^4^ (this issue is also addressed under *Synthesis and Chemical Stability*).

Using iodine-125 as a label comes along with some disadvantages: due to the short half-life (59.4 days) and high susceptibility to radiolysis, iodine-125-labeled radioligands can only be used over a period of a few months;^29,30^ special safety precautions during preparation and handling are needed; and the altered structure and physiochemical properties (increased lipophilicity), caused by iodination, can potentially affect the pharmacological profile of the ligand. Iodine-125 is commonly introduced at the tyrosine residues of the peptides. As the peptides of the NPY family include multiple tyrosine residues, labeling could occur at more than one tyrosine residue.^22,31^ Tritiated ligands are superior to iodine-125-labeled ligands since stocks can be stored and used for several years due to the longer half-life (12.3 years) of tritium. Furthermore, handling of tritium-labeled compounds is more convenient, and the hydrogen-tritium exchange has almost no impact on the physicochemical properties of the ligand.^30^ So far, tritiated derivatives of NPY ([Lys^4^-[^3^H]propionyl]pNPY,^28,32,33^*N*-[^3^H]propionyl-NPY^34^), but no tritium-labeled derivatives of PYY, have been reported. The tritiated pNPY derivative [Lys^4^-[^3^H]propionyl]pNPY shows a low binding affinity for the Y_2_R in sodium-containing buffer [studied at intact Chinese hamster ovary (CHO) cells stably expressing the hY_2_R],^35^ whereas *N*-[^3^H]propionyl-NPY could be successfully used for Y_2_R binding studies at calf hippocampus membrane preparations using a sodium-containing buffer (cf. Table 1).^34^ Generally, receptor–ligand binding studies should be performed using sodium-containing buffers (ca. 140–150 mM Na^+^) since sodium-free buffers, which have also been used for Y_2_R binding assays (cf. Table 1), do not represent physiological-like conditions.

Besides radiolabeled derivatives of NPY and PYY, which represent Y_2_R agonists, radiolabeled Y_2_R antagonists, exhibiting lower molecular weight compared to those of NPY and PYY, could also be used to determine Y_2_R binding affinities. However, to date, radiolabeled Y_2_R antagonists with high Y_2_R binding affinity (*K*_d_ < 10 nM) have not been reported. The only reported radiolabeled and pharmacologically characterized Y_2_R antagonists are two tritium-labeled ligands derived from the argininamide-type, Y_2_R selective antagonist BIIE0246 (Figure 2). These radioligands ([^3^H]UR-PLN196^44^ and [^3^H]UR-PLN208^45^) showed either insurmountable antagonism and pseudoirreversible binding^44^ at the Y_2_R or high unspecific binding and moderate chemical stability at pH 7.4,^45^ i.e., they do not represent ideal probes for Y_2_R binding studies.

**Figure 2:**
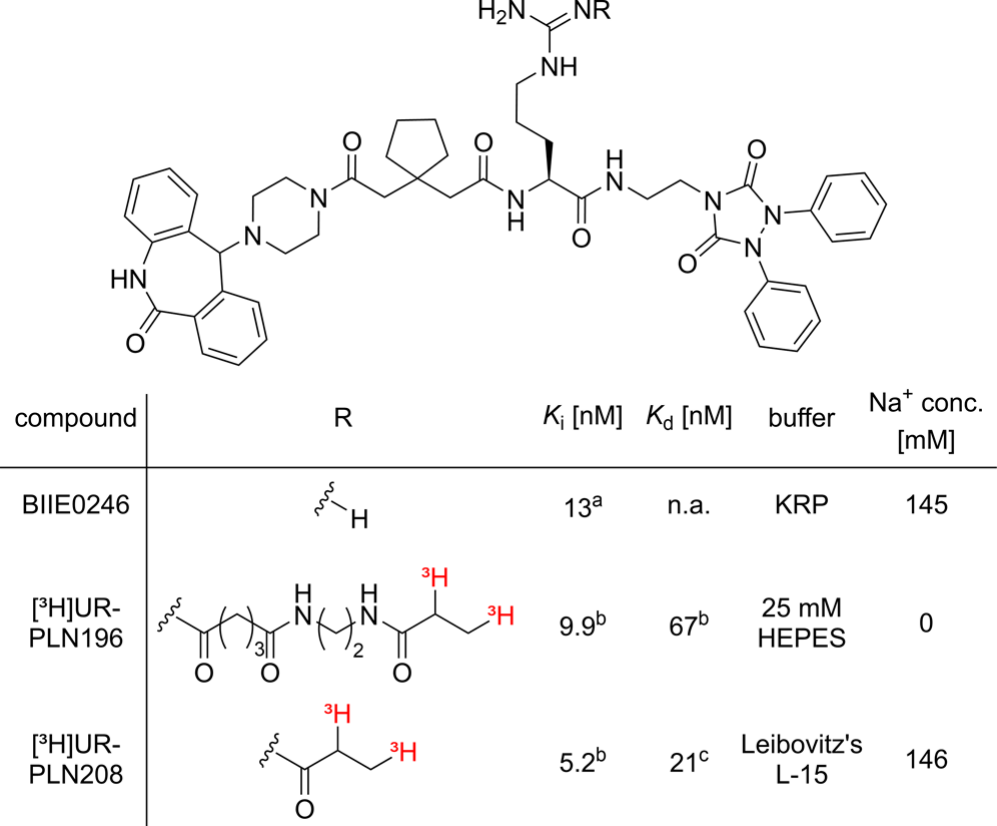
Structures of the reported BIIE0246-derived tritiated Y_2_R antagonists and their Y_2_R binding affinities. ^*a*^Dumont et al.^37^ The reported *K*_i_ value of BIIE0246 was calculated from the reported IC_50_ (IC_50_ = 15 nM, determined using [^125^I]PYY(3–36) (c_final_ = 32.5 pM, *K*_d_ = 0.25 nM) at HEK293-hY_2_R cells) using the Cheng–Prusoff equation.^46^^b^Pluym et al.^44^^c^Baumeister et al.^45^ n.a. not applicable.

Aiming for a tritiated, conveniently accessible Y_2_R radioligand that shows high binding affinity in sodium-containing buffer, a tritiated derivative of hPYY, containing, in contrast to hNPY, no oxidation prone methionine, was synthesized and characterized in sodium-free and sodium-containing buffer by Y_2_R saturation binding, Y_2_R binding kinetics, and competition binding with reference Y_2_R ligands.

## Results and Discussion

### Synthesis and Chemical Stability

The commercially available radiolabeling reagent succinimidyl [^3^H]propionate ([^3^H]**1**) has been used to prepare the tritiated pNPY derivative [^3^H]propionyl-pNPY via propionylation at Lys^4^.^28,43,47,48^ Since PYY contains one lysine at the same position as in NPY (cf. Figure 1), a tritium-labeled PYY analogue should be available by [^3^H]propionylation at this lysine residue. Based on the reported cryo-EM structures of the Y_2_R in complex with hNPY^49,50^ or hPYY(3–36),^51^ showing similar binding modes, the derivatization of Lys^4^ of hPYY should not lead to a strong decrease in binding affinity (Figure S1, Supporting Information).

**Scheme 1:**
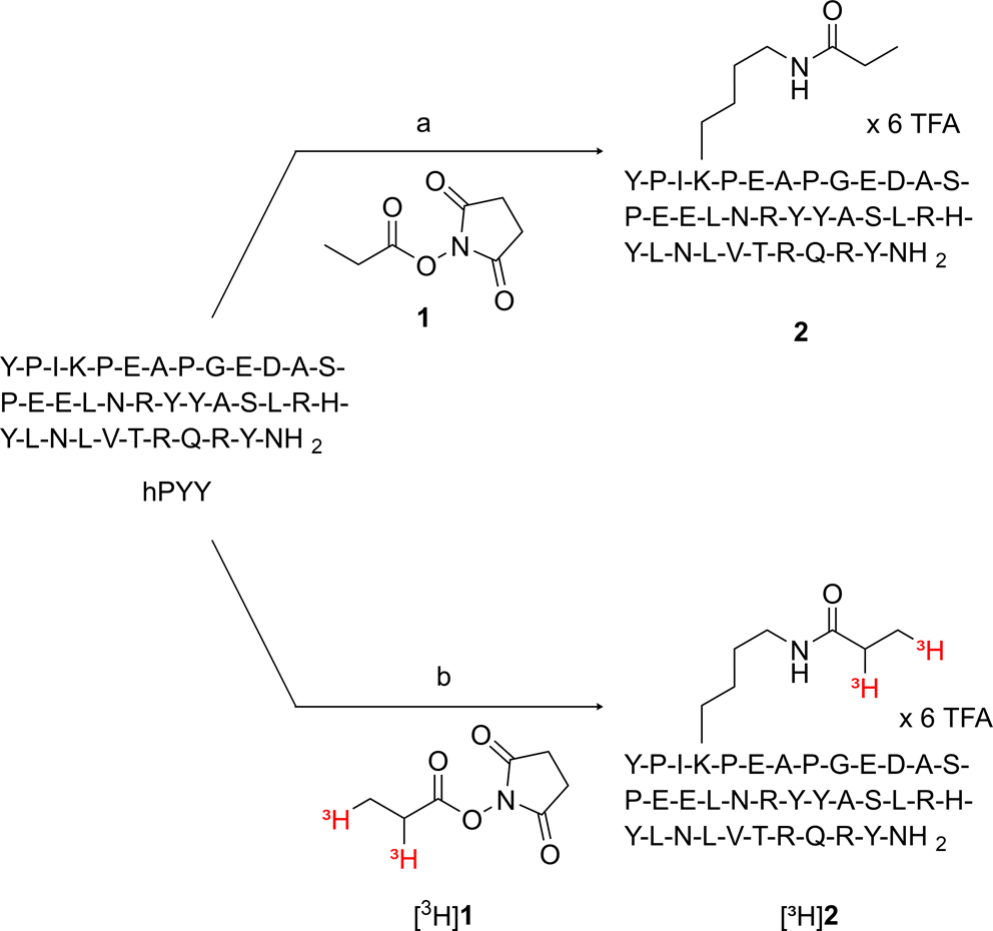
Synthesis of [Lys^4^-propionyl]hPYY (**2**) and [Lys^4^-[^3^H]propionyl]hPYY ([^3^H]**2**) Reagents and conditions: (a) DIPEA, DMF/NMP/H_2_O 60:25:15, rt, 1.5 h, 35%, (b) DIPEA, DMF/NMP/H_2_O 61:18:21, rt, 1.5 h, radiochemical yield: 25%. Note that the tritium atoms in the [^3^H]propionyl residue do not represent a quantity of tritium isotopes; they only indicate that tritium is present in the respective position.

Prior to the synthesis of [Lys^4^-[^3^H]propionyl]hPYY ([^3^H]**2**), the “cold” analogue [Lys^4^-propionyl]hPYY (**2**) was prepared by treatment of hPYY with succinimidyl propionate (**1**) in 1.5-fold excess over hPYY. This reaction, performed in a mixture of DMF, NMP, and water and in the presence of *N,N*-diisopropylethylamine (DIPEA), gave **2** in 35% yield (Scheme 1). The low yield can mainly be attributed to the formation of a double propionylated side product (the second propionyl group is most likely attached to the N-terminus of hPYY). It should be noted that the reaction was stopped before the entire starting material had been consumed (for a chromatogram of the HPLC analysis of the reaction mixture see Figure S2, Supporting Information). Moreover, the formation of aggregates of the peptide during purification by preparative HPLC can lower the yield.

As lysine is known to exhibit the second highest nucleophilic reactivity for acylating reagents after cysteine^52^ compared to the other proteinogenic amino acids, the propionylation of PYY, containing no cysteine, predominantly occurs at the lysine residue at position 4. In this context, it should be mentioned that in Czerwiec et al., the [^3^H]propionylated NPY derivative used for Y_2_R binding studies is denoted *N*-[^3^H]propionyl-NPY, indicating a propionylation at the *N*-terminus of NPY.^34^ As an N-terminal propionylation would require a protection of the ε-amino group of Lys^4^ in NPY during [^3^H]propionylation, we assume that Czerwiec et al. used [Lys^4^-[^3^H]propionyl]NPY for their studies.

As **2** was purified by preparative RP-HPLC using 0.1% aqueous TFA as the aqueous mobile phase, it was obtained as a TFA salt after lyophilization of the eluate. Generally, it can be assumed that the number of TFA molecules equals the number of basic amino acids. However, as the peptide sequence of hPYY also contains several acidic amino acids, possibly forming inter- or intramolecular salt bridges with the basic amino acids resulting in a release of TFA, the actual number of TFA^–^ counterions might be lower than the number of basic amino acids in the peptide. To investigate this issue, a fluorinated congener of PYY (compound **4**), containing a 2-fluoropropionyl moiety instead of the propionyl group in **2**, was synthesized as a reference compound (Scheme S1, Supporting Information; for details, see Experimental Section). Peptide **4** was isolated by preparative HPLC using the same conditions as those for the purification of **2**, presumably yielding the TFA salt of **4**. The integral ratio of the ^19^F signals of **4** and TFA^–^ in the ^19^F NMR spectrum was 1:20.4 (Figure S3, Supporting Information). This ratio indicates a number of six to seven TFA^–^ counterions. Due to the use of a very low sample concentration (4.9 mg/mL) for the measurement of the ^19^F NMR spectrum and the interference of the signal of the 2-fluoropropionyl group with the background signal arising from the polytetrafluoroethylene (PTFE)-containing probe head of the NMR spectrometer, the integral of the ^19^F signal of the 2-fluoropropionyl group might be biased. Therefore, we acquired a ^19^F NMR spectrum of a reference sample, containing 4-nitrophenyl 2-fluoropropanoate (**3**) and TFA in a precise ratio of 1:6 (the molar concentration of **3** corresponded approximately to the molar concentration of **2** used for the analysis of **2**). Integration of the ^19^F signals in the ^19^F spectrum of the reference sample yielded an integral ratio of 1:20.6 (Figure S4, Supporting Information). These data revealed that the PYY derivatives **2** and **4** were isolated as TFA salts containing six TFA^–^ counterions. This, in turn, means that the number of TFA^–^ counterions corresponds to the number of basic groups (N-terminus, Arg^19^, Arg^25^, His^26^, Arg^33^, and Arg^35^) in the peptides.

The investigation of the chemical stability of **2** in PBS (pH 7.4) at 24 °C over 24 h showed no decomposition (Figure S5, Supporting Information), indicating that **2** and [^3^H]**2** exhibit sufficiently high chemical stability for binding assays.

**Figure 3:**
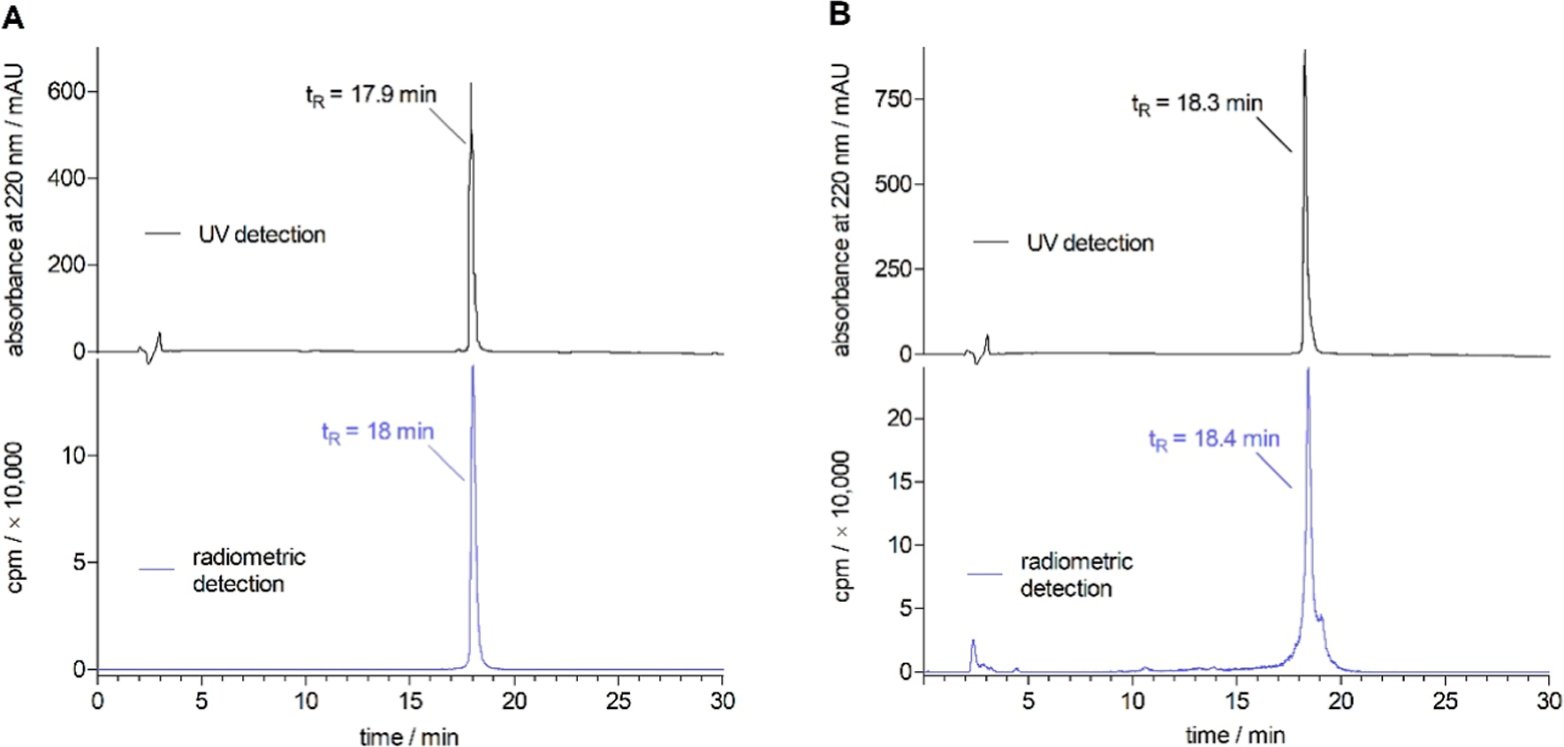
(A) Radiochemical purity (99%) of [^3^H]**2** directly after synthesis determined by RP-HPLC analysis. (B) Radiochemical purity (ca. 80%) of [^3^H]**2** determined 22 months after synthesis. Analyzed were solutions of [^3^H]**2** (0.24 μM) spiked with 19 μM **2** (injection volume: 100 μL).

For the synthesis of the radiolabeled peptide [^3^H]**2**, an excess of hPYY was treated with [^3^H]**1** in the presence of DIPEA (Scheme 1). [^3^H]**2** was isolated by RP-HPLC (allowing for a separation from the precursor hPYY; for a representative chromatogram see Figure S6, Supporting Information) to obtain the radioligand in high chemical purity (99%). After 22 months of storage at 4 °C in a weakly acidic aqueous solution, [^3^H]**2** still showed satisfactory radiochemical purity (≥80%) (Figure 3).

## YR Binding of **2**

To examine the Y_1_R, Y_2_R, Y_4_R, and Y_5_R binding of **2**, well-established competition binding assays for each receptor subtype were applied (Y_1_R: Keller et al.,^47^ Y_2_R: Konieczny et al.,^43^ Y_4_R: Gleixner et al.,^33^ Y_5_R: Kuhn et al.^53^). The obtained radioligand competition binding curves are depicted in Figure S7 (Supporting Information), and the p*K*_i_ values are shown in Table 2. Whereas the investigation of Y_1_R, Y_4_R, and Y_5_R binding was performed in sodium-containing buffers, Y_2_R binding affinities, using [Lys^4^-[^3^H]propionyl]pNPY as radioligand, were determined in sodium-free buffer (*buffer I*), as no saturation could be reached with this radioligand in saturation binding experiments using intact CHO-hY_2_R cells (Figure S8, Supporting Information). This is in agreement with previously reported results of saturation binding experiments performed with [Lys^4^-[^3^H]propionyl]pNPY at CHO-hY_2_-Gq_i5_-mtAEQ in a sodium-containing buffer.^35^

**Table 2:** YR Binding Data of hPYY and **2**

compd.	p*K*_i_ ± SEM/*K*_i_ [nM]			
	hY_1_R^a^	hY_2_R^b^	hY_4_R^c^	hY_5_R^d^
hPYY	8.91 ± 0.08/1.3	9.47 ± 0.04/0.34	6.69 ± 0.07/210	8.53 ± 0.15/3.3
**2**	8.38 ± 0.04/4.2	9.38 ± 0.05/0.43	6.56 ± 0.06/280	7.90 ± 0.15/13

a Determined by competition binding at Y_1_R-expressing SK-N-MC neuroblastoma cells using [^3^H]UR-MK299 (*K*_d_ = 0.058 nM, *c* = 0.075 nM) as radioligand.

b Determined by competition binding with [Lys^4^-[^3^H]propionyl]pNPY (*K*_d_ = 0.14 nM,^43^*c* = 0.5 nM) at CHO-hY_2_R cells.

c Determined by competition binding at CHO-hY_4_R-G_qi5_-mtAEQ cells using [^3^H]UR-JG102 (*K*_d_ = 0.11 nM,^33^*c* = 0.25 nM) as radioligand.

d Determined by competition binding at HEC-1B-hY_5_R cells using [Lys^4^-[^3^H]propionyl]pNPY (*K*_d_ = 11 nM,^28^*c* = 5 nM) as radioligand. Data represent mean values ± SEM (p*K*_i_) or mean values (*K*_i_) from three to five independent experiments performed in triplicate. Note: Whereas Y_1_R, Y_4_R, and Y_5_R affinities were determined in sodium-containing buffer, Y_2_R binding was studied in sodium-free buffer.

Comparison of the YR binding affinities of hPYY and **2** shows that the introduction of the propionyl group at Lys^4^ has no impact on Y_2_R and Y_4_R binding, but results in a low decrease in binding affinity in the case of the Y_1_R and Y_5_R (Table 2). This supports the abovementioned hypothesis that Lys^4^ of PYY does not undergo relevant interactions with the Y_2_R. Based on the data presented in Table 2, **2** displays moderate Y_2_R selectivity over the Y_1_R and Y_5_R (10-fold and 30-fold, respectively) and high selectivity over Y_4_R (650-fold), as also observed for hPYY. It should be emphasized that Y_2_R binding affinities were determined in sodium-free buffer (in contrast to Y_1_R, Y_4_R, and Y_5_R binding assays), potentially resulting in higher agonist binding affinity compared to sodium-containing buffer (see discussion below). Consequently, the Y_2_R selectivity of **2** could be lower when Y_2_R binding is determined in the presence of sodium.

The Y_2_R agonistic activity of **2** was studied in a Fura-2 Ca^2+^ assay using CHO-hY_2_R cells. Peptide **2** displayed full Y_2_R agonism relative to hPYY (concentration–response curves shown in Figure S9, Supporting Information).

### Y_2_R Binding Studies with [^3^H]2

All Y_2_R binding experiments with [^3^H]**2** were conducted with a suspension of CHO cells stably transfected with the gene encoding for the hY_2_R. For the determination of unspecific binding of [^3^H]**2** in all types of Y_2_R binding assays, an excess of nonpeptidic Y_2_R antagonist was used. Generally, the use of the “cold” analogue of the radioligand for the determination of unspecific binding should be avoided, as this can result in a marked displacement of the radiolabeled probe from unspecific binding sites. This would lead to the determination of false-specific binding in addition to true specific binding. This, in turn, would result in the determination of apparent *K*_d_ values being higher than the true *K*_d_. For this reason, unspecific binding of [^3^H]**2** was determined in the presence of the Y_2_R antagonists JNJ31020028^39^ and BIIE0246.^23^ A mixture of both antagonists was used to ensure an effective displacement of the radiolabeled peptide from Y_2_R (note: a radiolabeled derivative of BIIE0246 could not be effectively displaced from Y_2_R by pNPY questioning a competitive interaction between BIIE0246 and pNPY or PYY at Y_2_R^44^).

**Figure 4:**
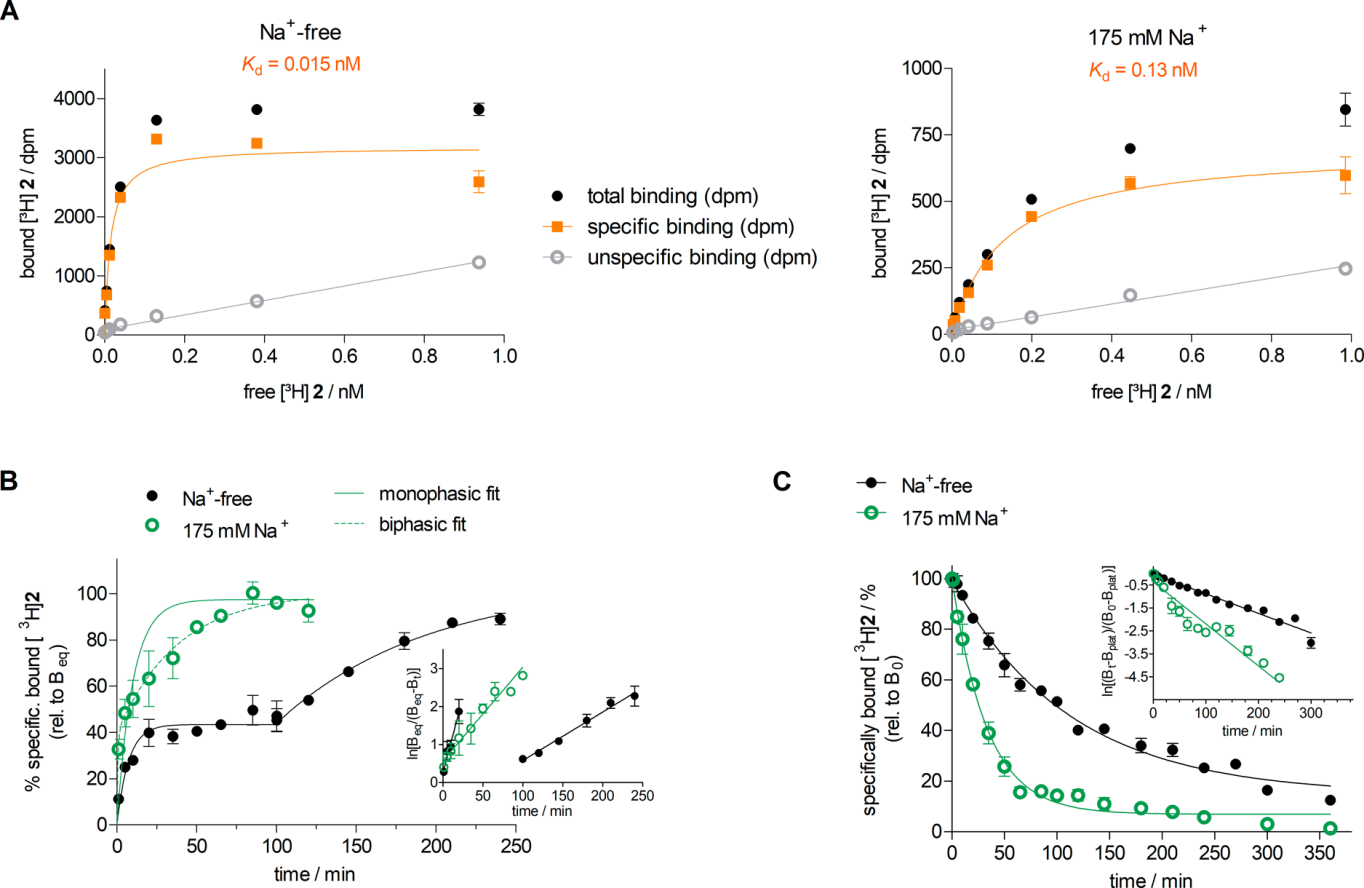
Y_2_R binding characteristics of [^3^H]**2** studied in sodium-free and sodium-containing buffer at 24 ± 2 °C using intact CHO-hY_2_R cells. (A) Representative Y_2_R binding isotherms (specific binding) of [^3^H]**2** obtained from saturation binding experiments performed in triplicate. Unspecific binding was determined in the presence of JNJ31020028 and BIIE0246 (1 μM each). Total and unspecific binding data represent mean values ± SEM. Specific binding data represent calculated values ± propagated error. (B) Association of [^3^H]**2** (*c* = 0.032 nM (Na^+^-free) or 0.32 nM (175 mM Na^+^)) to the Y_2_R. In contrast to sodium-containing buffer (*buffer II*), [^3^H]**2** exhibited a pronounced biphasic association kinetics in sodium-free buffer (*buffer I*). Proportion of fast/slow association components: 43:57 (*buffer I*), 60:40 (*buffer II*). Inset: linearized data. Data represent mean values ± SEM from three or five independent experiments each performed in triplicate. For *k*_obs_ and *k*_on_ values, see Table 3. (C) Dissociation of [^3^H]**2** from the Y_2_R. Concentration of [^3^H]**2** used for the preincubation (90 min): 0.16 nM (Na^+^-free) and 0.4 nM (175 mM Na^+^). Plateau values of the three-parameter fits (monophasic exponential decline): 15% (Na^+^-free) and 7% (175 mM Na^+^). Inset: linearized data. Data represent mean values ± SEM from four independent experiments each performed in triplicate. For *k*_off_ values, see Table 3.

Saturation binding was performed in sodium-free buffer (*buffer I*) and in sodium-containing buffer (*buffer II*, 175 mM Na^+^) (for detailed buffer compositions, see the Experimental Section). Initial saturation binding experiments with [^3^H]**2** were performed shortly after the synthesis of the radioligand, applying an incubation time of 2 h. In both buffers, the binding of [^3^H]**2** to the hY_2_R was saturable (Figure 4A), yielding apparent *K*_d_ values of 0.016 and 0.16 nM for *buffer I* and *buffer II*, respectively (Table 3). Unspecific binding at radioligand concentrations corresponding to the *K*_d_ value was <15% of total binding. The higher Y_2_R binding affinity of [^3^H]**2** in sodium-free buffer can most likely be attributed to the stabilization of the active receptor conformation in the absence of sodium, resulting in a tighter binding of receptor agonists.^54,55^ A systematic comparison of radioligand binding in sodium-free and sodium-containing buffer was also reported for the Y_4_R, which also revealed a higher binding affinity (10–20-fold) in sodium-free buffer.^28,33^

**Table 3:** Parameters Characterizing Y_2_R Binding of [^3^H]**2** Determined in Sodium-Free and Sodium-Containing Buffer at 24 ± 2 °C

buffer	saturation binding		binding kinetics			
	p*K*_d_/*K*_d_ [nM]^a^ initially determined	p*K*_d_/*K*_d_ [nM]^a^ after 2.5 years	*k*_obs_ [min^–1^]^b^	*k*_off_ [min^–1^]^c^	*k*_on_ [nM^–1^ min^–1^]^d^	*K*_d_ (kin) [nM]^e^
*buffer I* (sodium-free)	10.79 ± 0.001/0.016^f^	10.20 ± 0.01/0.063^f^	*k*_obs(bi,fast)_: 0.15 ± 0.03	0.0093 ± 0.0009	*k*_on(bi,fast)_: 4.3 ± 0.9	*K*_d_(kin)_bi,fast_: 0.0022 ± 0.0006
		10.17 ± 0.006/0.067^g^	*k*_obs(bi,slow)_: 0.015 ± 0.002		*k*_on(bi,slow)_: 0.18 ± 0.10	*K*_d_(kin)_bi,slow_: 0.05 ± 0.03
*buffer II* (175 mM Na^+^)	9.80 ± 0.02/0.16^f^	9.75 ± 0.04/0.18^f^	*k*_obs,mono_: 0.11 ± 0.05	0.033 ± 0.004	*k*_on,mono_: 0.24 ± 0.18	*K*_d_(kin)_mono_: 0.14 ± 0.11
			*k*_obs(bi,fast)_: 1.8 ± 0.3		*k*_on(bi,fast)_: 5.57 ± 0.96	*K*_d_(kin)_bi,fast_: 0.006 ± 0.002
			*k*_obs(bi,slow)_: 0.029 ± 0.004		*k*_on(bi,slow)_: –0.01 ± 0.03	*K*_d_(kin)_bi,slow_: n.a.

a Equilibrium dissociation constant expressed as p*K*_d_ (mean values ± SEM) and *K*_d_ (mean values) obtained from 3 to 11 independent experiments (performed in triplicate).

b Observed association rate constants obtained by monophasic or biphasic fitting. Mean values ± SEM from three or five independent experiments (performed in triplicate).

c Dissociation rate constants obtained from three-parameter monophasic fits (exponential decline). Mean values ± SEM from four independent experiments (performed in triplicate).

d Association rate constant ± propagated error calculated from *k*_obs_, *k*_off_, and the ligand concentration used for the association studies.

e Kinetically derived dissociation constants ± propagated error calculated from *k*_on_ and *k*_off_ values.

f Determined with an incubation time of 2 h.

g Determined with an incubation time of 4.5 h. n.a. not applicable.

Saturation binding experiments were repeated under the same conditions about 2.5 years after the synthesis of [^3^H]**2**. For the sodium-containing *buffer II*, the initially determined *K*_d_ value of 0.16 nM could be well reproduced (*K*_d_ = 0.18 nM) confirming the high stability of the radioligand [^3^H]**2**. In the case of *buffer I*, additional saturation binding experiments with an incubation time of 4.5 h were performed, because the association kinetics (presented below) of [^3^H]**2** in *buffer I* indicated that equilibrium is not reached in less than 4 h. These studies yielded apparent *K*_d_ values of 0.063 nM and 0.067 nM for an incubation time of 2 and 4.5 h, respectively, showing that the difference in the incubation time had no significant effect on the determined *K*_d_. However, in contrast to *buffer II*, the initially determined *K*_d_ of 0.016 nM could not be well reproduced in the case of *buffer I* (cf. Table 3). Possibly, the differences in *K*_d_ values (initially determined vs redetermined after 2.5 years) can be attributed to very little variations in the composition of the sodium-free buffer, effecting receptor-agonist binding, or to slight variations in the cell harvest procedure (although performed according to the same protocol), resulting in different amounts of residual sodium. These results show that the binding assay under sodium-free conditions is less robust than the assay performed in sodium-containing buffer.

The association and dissociation kinetics of [^3^H]**2** were also studied in *buffer I* and *buffer II*. Analysis of the data obtained from the association experiments in *buffer II* with a monophasic and biphasic exponential fit revealed a biphasic character of the association (two-phase association favored over one-phase association according to the *F*-test, *P* value < 0.0001, GraphPad Prism 5). The proportion of the fast and slow association components was 60:40 (Figure 4B). Under sodium-free conditions (*buffer I*), plateauing of the initial association phase was more pronounced. In contrast to the association data determined in *buffer II*, these data could not be fitted with the two-phase association fit (ambiguous results, GraphPad Prism 5, Figure S10, Supporting Information). Therefore, as an approximation, the data determining the initial association phase and the data largely defining the second association phase were analyzed separately according to equations describing a monophasic association (in the case of the second phase, starting from the plateau reached within the initial association phase; Figure 4B). The proportion of the fast and slow association components was 43:57.

The observed association rate constants *k*_obs_ and calculated association rate constants *k*_on_ are summarized in Table 3. In the case of association experiments performed in sodium-containing *buffer II*, the *k*_obs(bi,slow)_ value was lower than the *k*_off_ value obtained from dissociation experiments, resulting in a negative *k*_on(bi,slow)_ value, and, consequently, precluding a calculation of the kinetically derived dissociation constant *K*_d_(kin). Therefore, the data from the association studies in *buffer II* were additionally fitted with a monophasic exponential fit covering the whole association process (obtained *k*_obs,mono_ values and *k*_on,mono_ values are included in Table 3). A possible explanation for the biphasic association of [^3^H]**2** is the existence of two subpopulations of Y_2_R, one coupled to and the other uncoupled from the G protein, possibly also due to receptor overexpression in the cells. This is supported by the proportions of the fast and slow association components being similar for both binding buffers (*buffer I*: 43:57, *buffer II*: 60:40). Furthermore, it is supported by recent binding studies performed with radiolabeled and fluorescently labeled peptidic agonists of the neurotensin receptor 1 also using genetically engineered CHO cells.^56^ In case the receptor subpopulations correspond to G-protein coupling and noncoupling receptors, the initial fast association phase should predominantly originate from the binding of the radioligand to the G-protein-free receptor population, which is generally characterized by a more open passage to the ligand binding pocket.^57^ Accordingly, the second slow association phase should primarily represent binding to the population of receptors that can bind G-proteins. For the initial association phase, the absence or presence of sodium had almost no effect on the *k*_on(bi,fast)_ values (*buffer I*: 4.3 nM^–1^ min^–1^, *buffer II*: 5.6 nM^–1^ min^–1^). Concerning the second association phase, it is difficult to evaluate the effect of sodium since a negative value was obtained for *k*_on(bi,slow)_ (cf. Table 3). From the association curves, it is obvious that the absence of sodium causes an apparent delay of the second association phase compared to the association kinetics determined in sodium-containing buffer (Figure 4B). This might be explained by a more facile adaptation of the active receptor conformation in the absence of sodium and by the interaction with G-protein being characterized by a restricted access or narrowed passage to the ligand binding site.^54,55,57^

To investigate the dissociation of [^3^H]**2** from Y_2_R, an excess of the Y_2_R antagonists BIIE0246 and JNJ31020028 was added after preincubation of the CHO-hY_2_R cells with [^3^H]**2** for 90 min. For both conditions (sodium-free and sodium-containing buffer), the dissociation closely followed a monophasic exponential course (Figure 4C). To note, the analysis of the data using a biphasic fit (two phase decay, GraphPad Prism 5) failed. The dissociation of [^3^H]**2** in sodium-containing buffer (*buffer II*) was approximately 3.5 times faster compared to the sodium-free conditions (*k*_off_ = 0.033 min^–1^ vs 0.0093 min^–1^), being consistent with the lower binding affinity of [^3^H]**2** in *buffer II* compared to *buffer I* (Table 3). The slower dissociation in the sodium-free buffer can be explained by the stabilization of the active receptor conformation in the absence of sodium, featuring a more contracted ligand binding pocket than the inactive receptor conformation.^54,55^ The plateau values of the dissociation curves (three-parameter exponential fit, one phase decay, GraphPad Prism 5) were low (Na^+^-free: 15%, 175 mM Na^+^: 7%), but significantly different from zero (*P* < 0.05, one-tailed *t*-test). Low plateau values indicate an almost complete dissociation of the labeled ligand, a favorable feature with respect to the determination of receptor binding affinities of unlabeled ligands in competition binding assays.

Based on the data obtained from experiments performed in *buffer I*, the kinetically derived dissociation constant *K*_d_(kin) of [^3^H]**2** was calculated from *k*_on(bi,fast)_ and *k*_off_, and from *k*_on(bi,slow)_ and *k*_off_ according to the equation *K*_d_(kin) = *k*_off_/*k*_on_, yielding *K*_d_(kin) values of 0.0022 and 0.05 nM, respectively (cf. Table 3). The obtained *K*_d_ values of [^3^H]**2** from saturation binding experiments (*K*_d_ = 0.016 nM, 0.063 and 0.067 nM) correspond better to the *K*_d_(kin) derived from *k*_on(bi,slow)_. This shows that a calculation of *K*_d_(kin) based on *k*_on(bi,fast)_ implies an overestimation of the association rate, resulting in a decreased *K*_d_(kin) value. In the case of the sodium-containing *buffer II*, the *K*_d_(kin) value was calculated from *k*_on,mono_ and *k*_off_, and from *k*_on(bi,fast)_ and *k*_off_, affording values of 0.14 and 0.006 nM, respectively. The *K*_d_(kin) value of 0.14 nM, for which the entire association process is considered, was in excellent agreement with the *K*_d_ value of [^3^H]**2** obtained from saturation binding experiments in *buffer II* (*K*_d_ = 0.16 nM). In contrast, the *K*_d_(kin) value calculated from *k*_on(bi,fast)_ and *k*_off_ was considerably lower than the *K*_d_ value from saturation binding studies (0.006 nM vs 0.16 nM) due to negligence of the second slow association phase, meaning an overestimation of the association rate.

**Figure 5:**
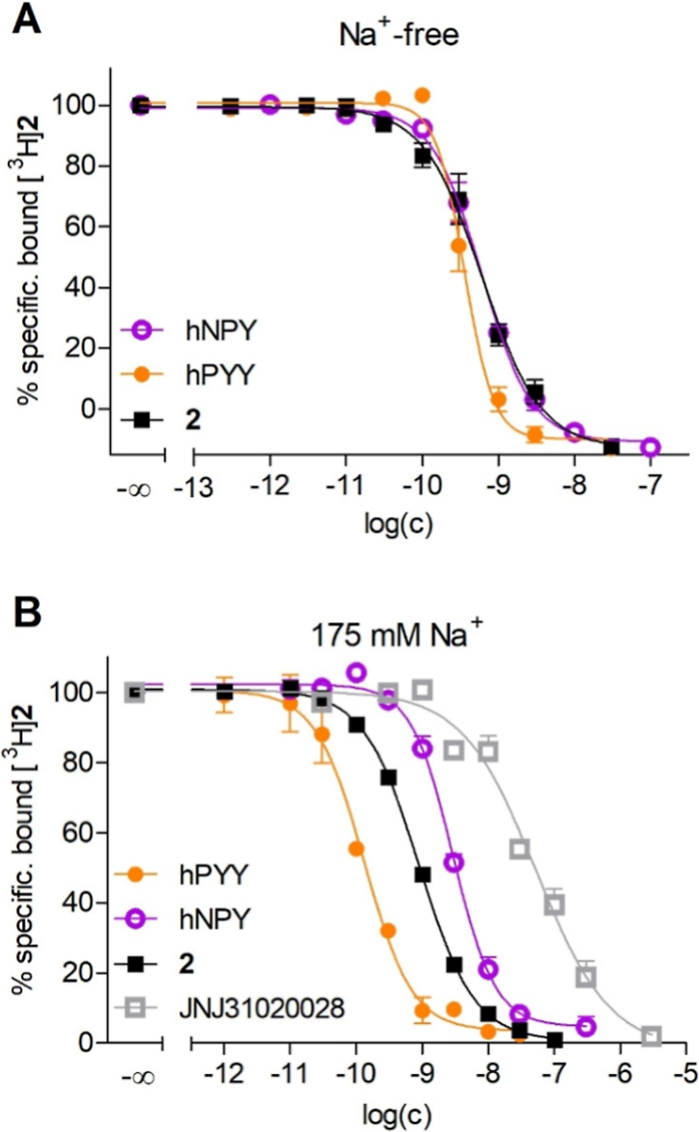
Displacement curves from a radiochemical Y_2_R competition binding assay performed with [^3^H]**2** and hPYY, hNPY, **2,** or JNJ31020028 at intact CHO-hY_2_R cells using a sodium-free (A) or sodium-containing (B) buffer. Data represent mean values ± SEM from at least three independent experiments performed in triplicate. *K*_i_ values are summarized in Table 4.

Finally, the suitability of [^3^H]**2** to serve as a molecular tool for the determination of the Y_2_R binding affinities of Y_2_R ligands was investigated. For this purpose, the *K*_i_ values of the peptidic ligands hPYY, hNPY, **2**, and the Y_2_R antagonist JNJ31020028 were determined in competition binding experiments at CHO-hY_2_R cells using [^3^H]**2** as probe (for radioligand displacement curves and *K*_i_ values, see Figure 5 and Table 4, respectively).

**Table 4:** Y_2_R Binding Affinities of hPYY, hNPY, **2**, and JNJ31020028 Determined by Competition Binding with [^3^H]**2**

compd.	sodium-free buffer		sodium-containing buffer	
	p*K*_i_/*K*_i_ [nM]^a^	literature (*K*_i_ in nM)	p*K*_i_/*K*_i_ [nM]^a^	literature (*K*_i_ or IC_50_ in nM)
hPYY	10.09 ± 0.06/0.089	0.14^b^	10.4 ± 0.04/0.040	*K*_i_: 0.34^c^ IC_50_: 0.30^d^
hNPY	9.84 ± 0.05/0.15	0.25^b^	9.11 ± 0.08/0.80	*K*_i_: 0.53^c^ IC_50_: 3.7^d^ IC_50_: 0.04^e^
**2**	9.84 ± 0.04/0.16	n.a.	9.59 ± 0.03/0.26	n.a.
JNJ31020028	n.d.	n.a.	7.87 ± 0.09/14.0	IC_50_ = 8.5^f^ IC_50_ = 6^g^

a Determined by competition binding at CHO-hY_2_R cells using [^3^H]**2** as a radioligand; mean values ± SEM (p*K*_i_) or mean values (*K*_i_) from three (hPYY, hNPY, and JNJ31020028 with Na^+^), four (hPYY, hNPY, and **2** Na^+^-free), or five (**2** with Na^+^) independent experiments performed in triplicate.

b Gehlert et al.^3^

c Gerald et al. (the reported p*K*_i_ value was converted to a *K*_i_ value).^24^

d Bonaventure et al. (species of the peptide was not indicated; the reported pIC_50_ value was converted to an IC_50_ value).^40^

e Cabrele et al.^58^

f Shoblock et al. (the reported p*K*_i_ value was converted to *K*_i_ value).^39^

g Swanson et al.^59^ n.d. not determined, n.a. not available.

The *K*_i_ values obtained for hPYY and hNPY in sodium-free buffer (*buffer I*) were in good agreement with the reported Y_2_R binding affinities of these peptides also determined in a sodium-free buffer (Table 4). The Y_2_R binding affinity of **2** (*K*_i_ = 0.16 nM) was slightly lower than the *K*_i_ value determined with [Lys^4^-[^3^H]propionyl]pNPY (*K*_i_ = 0.43 nM, cf. Table 1) and 2.4–10-fold higher than the apparent *K*_d_ values of [^3^H]**2** determined in the sodium-free buffer (cf. Table 3).

Regarding the *K*_i_ values determined in sodium-containing buffer (*buffer II*), the comparison of the *K*_i_ values of hPYY and hNPY with literature data is challenging since the described binding affinities of hNPY vary considerably, and for both peptides, some binding data are reported only as IC_50_ values (Table 4). The *K*_i_ of hPYY was moderately lower (<10-fold) compared to reported binding data, and the *K*_i_ of hNPY was in accordance with the reported *K*_i_ value of 0.53 nM (Table 4). Notably, the *K*_i_ obtained for **2** was in good agreement with the *K*_d_ value of [^3^H]**2** determined by saturation binding in sodium-containing buffer (*K*_i_ = 0.26 nM, *K*_d_ = 0.16 nM, cf. Tables 3 and 4). A *K*_i_ value of 0.26 nM also confirms the aforementioned Y_2_R selectivity of **2** over the other YR subtypes (cf. Table 1). The *K*_i_ value determined for the Y_2_R antagonist JNJ31020028 was in good agreement with reported IC_50_ values, showing that the peptidic radioligand [^3^H]**2** can be used to determine Y_2_R binding affinities of nonpeptidic Y_2_R antagonists targeting the orthosteric binding site.

## Conclusions

Propionylation of hPYY at Lys^4^ using succinimidyl [^3^H]propionate ([^3^H]**1**) afforded a tritiated derivative of hPYY ([^3^H]**2**) that shows saturable binding at CHO-hY_2_R cells (*K*_d_ = 0.16 nM) and favorable binding kinetics in a sodium-containing buffer. The suitability of [^3^H]**2** for the determination of Y_2_R binding affinities of Y_2_R ligands under physiological-like conditions (sodium-containing buffer) was demonstrated. To our best knowledge, this is the first study reporting the synthesis and pharmacological characterization of a tritium-labeled PYY derivative. [^3^H]**2** is superior to previously described [^3^H]propionylated NPY derivatives in terms of Y_2_R binding studies in sodium-containing buffer as binding of the latter to CHO-hY_2_R cells was not saturable. Furthermore, [^3^H]**2** is favored over ^125^I-labeled PYY or NPY derivatives with respect to safety precautions, production of a uniform radiolabeled species, stability, and long-term usage. Although [Lys^4^-propionyl]hPYY (**2**) binds with lower affinity to Y_5_R than to Y_2_R (*K*_i_ = 13 nM vs 0.43 nM), its tritiated analogue [^3^H]**2** could also serve as a radioligand for binding studies at Y_5_R for which, in contrast to Y_1_R, tritiated radioligands with high affinity are lacking. Therefore, the new radioligand represents a molecular tool useful for pharmacological research and drug screening related to the YR family. Moreover, the results suggest that fluorescence labeling of hPYY at Lys^4^ might give access to fluorescently labeled PYY derivatives, which can be used in fluorescence-based Y_2_R binding assays.

## Experimental Section

### Materials

NMP, DMF, TFA, and Pluronic F-127 were purchased from ACROS/Fisher Scientific (Schwerte, Germany). DIPEA was obtained from TCI (Eschborn, Germany). Acetonitrile (HPLC gradient grade) was obtained from VWR (Ismaning, Germany). Human peptide YY (hPYY), human neuropeptide Y (hNPY), porcine neuropeptide Y (pNPY), and human pancreatic polypeptide (hPP) were purchased from Synpeptide (Shanghai, China). Bacitracin, HEPES, and bovine serum albumin (BSA) were obtained from Serva (Heidelberg, Germany). Sigmacote and Fura-2 AM were obtained from Sigma-Aldrich (Taufkirchen, Germany). Fetal bovine serum (FBS) was purchased from Pan Biotech (Aidenbach, Germany). Succinimidyl [^3^H]propionate (molar activity: 3.44 TBq/mmol) was purchased from Novandi (Södertälje, Sweden). The Y_2_R antagonist BIIE0246 was obtained from Boehringer Ingelheim (Ingelheim, Germany), and JNJ31020028 was purchased from Cayman Chemical (Ann Arbor, USA). The syntheses of the radiolabeled Y_4_R ligand [^3^H]UR-JG102 (molar activity: 3.44 TBq/mmol)^33^ and 4-nitrophenyl 2-fluoropropanoate (**3**)^60^ were described previously. [Lys^4^-[^3^H]propionyl]pNPY (molar activity: 1.39 TBq/mmol) was prepared according to a previously reported procedure^47^ with minor modifications that were described elsewhere.^48^ The radiolabeled Y_1_R antagonist [^3^H]UR-MK299 (molar activity: 3.885 TBq/mmol) and succinimidyl propionate (**1**) were prepared according to reported procedures.^47^ Millipore water was consistently used for the preparation of stock solutions, buffers, and aqueous eluents for HPLC. Polypropylene reaction vessels (1.5 and 2 mL) from Sarstedt (Nümbrecht, Germany) were used to prepare and keep stock solutions for stability studies and as reaction vessels for the synthesis of **2** and [^3^H]**2**.

### Mass Spectrometry

High-resolution mass spectrometry (HRMS) was performed with an Agilent 6540 UHD accurate-mass Q-TOF LC/MS system coupled to an Agilent 1290 analytical HPLC system (Agilent Technologies. Santa Clara, CA) using an ESI source and the following LC method: column: Luna Omega C18, 1.6 μm, 50 × 2.1 mm (Phenomenex, Aschaffenburg, Germany), column temperature: 40 °C, solvent/linear gradient: 0–4 min: 0.1% aqueous HCOOH/acetonitrile supplemented with 0.1% HCOOH 95:5–2:98, 4–5 min: 2:98, flow: 0.6 mL/min.

### Preparative HPLC

Preparative HPLC was performed with a system from Knauer (Berlin, Germany) consisting of two K-1800 pumps and a K-2001 detector. A Gemini NX-C18, 5 μm, 250 mm × 21 mm (Phenomenex) was used as a reversed-phase (RP) column at a flow rate of 20 mL/min using mixtures of 0.1% aqueous TFA and acetonitrile as the mobile phase. A detection wavelength of 220 nm was used throughout. Collected fractions were lyophilized using a Scanvac CoolSafe 100–9 freeze-dryer (Labogene, Allerød, Denmark) equipped with an RZ 6 rotary vane vacuum pump (Vacuubrand, Wertheim, Germany).

### Analytical HPLC

Analytical HPLC analysis was performed with a 1100 series system from Agilent Technologies composed of a degasser (G1379A), a binary pump (G1312A), a variable wavelength detector (G1314A), a thermostated column compartment (G1316A), and an autosampler (G1329A). Detection was performed at 220 nm, and the oven temperature was 30 °C. For the analysis of reaction mixtures and purity controls, a Kinetex-XB C18 100A, 5 μm, 250 × 4.6 mm (Phenomenex) served as a stationary phase at a flow rate of 0.8 mL/min. Mixtures of 0.05% aqueous TFA (A) and acetonitrile (B) were used as the mobile phase. The following linear gradient was applied: 0–30 min: A/B 90:10–5:95, 30–40 min: 5:95 (isocratic). The injection volume was 80 μL. For the analysis of the samples from stability studies with **2** in PBS, a Gemini-NX C18, 100A, 5 μm, 250 × 4.6 mm (Phenomenex) served as stationary phase at a flow rate of 0.8 mL/min. Mixtures of 0.25% aqueous TFA (C) and 0.175% TFA in acetonitrile (D) were used as the mobile phase. The following linear gradient was applied: 0–25 min: C/D 90:10–5:95, 25–32 min: 5:95 (isocratic). The injection volume was 40 μL. Retention (capacity) factors *k* were calculated from the retention times *t*_R_ according to *k* = (*t*_R_ – *t*_0_)/*t*_0_ (*t*_0_ = dead time, 2.6 min for the system, column and flow rate mentioned above).

## Syntheses Protocols and Analytical Data

### Tyr-Pro-Ile-*N*^ε^-propionyl-Lys-Pro-Glu-Ala-Pro-Gly-Glu-Asp-Ala-Ser-Pro-Glu-Glu-Leu-Asn-Arg-Tyr-Tyr-Ala-Ser-Leu-Arg-His-Tyr-Leu-Asn-Leu-Val-Thr-Arg-Gln-Arg-Tyr-amide hexakis(hydrotrifluoroacetate) (**2**)

The reaction was carried out in a 1 mL reaction vessel. A solution of *N*-succinimidyl propionate (**1**) (0.45 mg, 2.6 μmol) in anhydrous DMF (3.2 μL) was added in two portions (1.6 + 1.6 μL with a time lag of 4 min) to a stirred solution of hPYY (9.0 mg, 1.8 μmol) and DIPEA (4.3 μL, 24.7 μmol) in DMF/NMP/H_2_O 60:25:15 (100 μL). The mixture was stirred at rt for 3 h. 10% aqueous TFA (19 μL) was added followed by isolation of the product by preparative HPLC (gradient: 0–30 min: 0.1% aqueous TFA/acetonitrile 80:20–50:50, *t*_R_ = 13 min) yielding **2** as a white fluffy solid (3.1 mg, 35%). HRMS (ESI): *m*/*z* [M+4H]^4+^ calcd. for [C_197_H_303_N_55_O_58_]^4+^ 1092.3124, found: 1092.3136. RP-HPLC (220 nm): > 99% (*t*_R_ = 12.7 min, *k* = 3.9). C_197_H_299_N_55_O_58_•C_12_H_6_F_18_O_12_ (4365.89 + 684.14).

### Tyr-Pro-Ile-*N*^ε^-(2-fluoropropionyl)-Lys-Pro-Glu-Ala-Pro-Gly-Glu-Asp-Ala-Ser-Pro-Glu-Glu-Leu-Asn-Arg-Tyr-Tyr-Ala-Ser-Leu-Arg-His-Tyr-Leu-Asn-Leu-Val-Thr-Arg-Gln-Arg-Tyr-amide hexakis(hydrotrifluoroacetate) (**4**)

The reaction was carried out in a 1 mL reaction vessel. A solution of 4-nitrophenyl 2-fluoropropanoate (**3**) (0.71 mg, 3.4 μmol) in anhydrous DMF (5.46 μL) was added in two portions (2.73 + 2.73 μL with a time lag of 2 min) to a stirred solution of hPYY (11.4 mg, 2.23 μmol) and DIPEA (5.44 μL, 31.3 μmol) in DMF/H_2_O 80:20 (200 μL). The mixture was stirred at rt for 1.5 h. 10% aqueous TFA (20 μL) was added followed by isolation of the product by preparative HPLC (gradient: 0–25 min: 0.1% aqueous TFA/acetonitrile 90:10–50:50, *t*_R_ = 16.9 min) yielding **3** as a white fluffy solid (2.1 mg, 18%). HRMS (ESI): *m*/*z* [M+4H]^4+^ calcd. for [C_197_H_302_FN_55_O_58_]^4+^ 1096.8101, found: 1096.8115. RP-HPLC (220 nm): > 99% (*t*_R_ = 12.9 min, *k* = 4.0). C_197_H_298_FN_55_O_58_•C_12_H_6_F_18_O_12_ (4383.88 + 684.14).

#### Synthesis of [^3^H]**2**

The radioligand [^3^H]**2** was prepared following a reported procedure for the syntheses of [^3^H]propionylated neurotensin receptor ligands, which was modified as required.^56^ A solution of succinimidyl [^3^H]propionate (molar activity: 3.44 TBq/mmol, purchased from Novandi, Södertälje, Sweden) (87.9 MBq, 1.25 mL, 25.5 nmol) in *n*-heptane/EtOAc 3:2 v/v was transferred from the delivered ampule into a 1.5 mL polypropylene reaction vessel with a screw cap, and the solvent was removed in a vacuum concentrator (rt, ca. 50 min). A solution of hPYY (0.42 mg, 82.2 nmol) and DIPEA (1.8 μL, 10.3 μmol) in DMF/NMP/H_2_O 61:18:21 v/v (45 μL) was added, immediately followed by vortexing. Subsequently, the vessel was shaken at rt for 1.5 h. The mixture was acidified by the addition of 2% aqueous TFA (65 μL), followed by the addition of 1:9 v/v acetonitrile/0.05% aqueous TFA (200 μL) and H_2_O (150 μL). As this gave a cloudy mixture, 2% aqueous TFA (40 μL) and acetonitrile/H_2_O 8:2 v/v (60 μL) were added. This resulted in a reduction but not in a complete dissolving of insoluble material. The slightly cloudy solution was directly injected into the HPLC system (no filtration). [^3^H]**2** was isolated using an HPLC system from Waters (Eschborn, Germany) consisting of two pumps 510, a pump control module, a manual injector (loop size: 200 μL), a 486 UV/vis detector, and a Flow-one Beta series A-500 radiodetector (Packard, Meriden, USA) (the latter was disconnected during the purification process, i.e., fractions containing [^3^H]**2** were collected based on UV detection). A Luna C18(2) column (3 μm, 150 × 4.6 mm, Phenomenex, Aschaffenburg, Germany) was used as the stationary phase at a flow rate of 0.8 mL/min. Mixtures of 0.05% aqueous TFA (A) and acetonitrile supplemented with 0.04% TFA (B) were used as the mobile phase. The following linear gradient was applied: 0–20 min: A/B 80:20–66:34, 20–22 min: 66:34–5:95, 22–28 min: 5:95 (isocratic). Four runs were conducted (for a representative chromatogram see Figure S6, Supporting Information). All fractions containing [^3^H]**2** (*t*_R_ = 21.0 min) were collected and combined in a 2 mL polypropylene reaction vessel with a screw cap. The volume of the combined eluates was reduced by evaporation in a vacuum concentrator to approximately 610 μL. Ethanol (68 μL) and a 1:9 v/v mixture of ethanol/water (123 μL) were added, resulting in a mixing ratio of EtOH/aqueous solvent 1:9 v/v and a total volume of 800 μL (preliminary stock solution). To determine the radiochemical purity and to prove the identity of [^3^H]**2**, 3 μL of the preliminary stock solution were added to 100 μL of a 20 μM solution of hPYY in acetonitrile/0.05% aqueous TFA 1:9 v/v, affording the sample to be analyzed using the aforementioned HPLC system, column, and solvents (injection volume: 100 μL). The following linear gradient was applied: 0–20 min: A/B 85:15–58:42, 20–32 min: 58:42–5:95, 32–38 min: 5:95 (isocratic). The radiochemical purity was >99% (*t*_R_ = 18.0 min). To quantify the activity of [^3^H]**2** and to determine the molar concentration, 2 × 2 μL of the preliminary stock solution were added to 998 μL of DMSO/H_2_O 8:2 v/v and 4 × 10 μL of these dilutions were counted in 3 mL of liquid scintillator (Rotiscint Eco Plus, Carl Roth, Karlsruhe, Germany) with a Tri-Carb 3100TR liquid scintillation counter (PerkinElmer). The activity concentration was adjusted to 15.0 MBq/mL by the addition of 1:9 v/v ethanol/H_2_O (681 μL), resulting in a final concentration of 4.36 μM and a total volume of 1474 μL. Radiochemical yield: 22.11 MBq (0.598 mCi), 25.2%. Molar activity: as the supplier of the labeling reagent succinimidyl [^3^H]propionate (Novandi, Södertälje, Sweden) provides a precisely determined molar activity and due the fact that [^3^H]**2** bears exactly one tritiated propionyl residue originating from the labeling reagent, the molar activity of [^3^H]**2** was defined to be equal to the molar activity of the labeling reagent, amounting to 3.44 TBq/mmol (it is assumed that under the mild reaction conditions the carbon-tritium bonds remained intact). The molar activity of the labeling reagent succinimidyl [^3^H]propionate ([^3^H]**1**) was determined by LC–MS analysis. Coinjection of “cold” succinimidyl propionate allowed quantification of the incorporated tritium (Novandi, Södertälje, Sweden).

## Chemical Stability

The chemical stability of peptide **2** was investigated in PBS (adjusted to pH 7.4) at 24 °C using siliconized (Sigmacote, Sigma) 1 mL polypropylene reaction vessels. The incubation was started by the addition of 2.4 μL of a 5 mM stock solution (solvent: 10 mM HCl) to 117.6 μL of PBS to yield a concentration of 100 μM. After time periods of 0, 6, and 24 h, an aliquot (30 μL) was withdrawn and added to 30 μL of acetonitrile/1% aq. TFA 1:1 (v/v) to obtain a peptide solution with a concentration of 50 μM. 40 μL of this solution were subjected to analytical RP-HPLC analysis. Under the same conditions, neat PBS was kept and treated in a separate vessel and was diluted and analyzed as the samples containing **2** to obtain blank chromatograms (vehicle controls) for 0 and 24 h.

## Cell Culture

Cell culture conditions for SK-N-MC neuroblastoma cells (obtained from the American Type Culture Collection, ATCC HTB-10), CHO-hY_2_R cells (obtained from PerkinElmer, Rodgau, Germany), CHO-hY_4_-Gq_i5_-mtAEQ,^61^ and HEC-1B-hY_5_R cells^62^ were described previously.^33^

## Buffers Used for Binding and Functional Assays

*Buffer I* (used for binding experiments at the Y_2_R): hypotonic sodium-free HEPES buffer (25 mM HEPES, 2.5 mM CaCl_2_, 1 mM MgCl_2_, adjusted to pH 7.4 using 25% ammonia in water) supplemented with 1% BSA (Serva, Heidelberg, Germany) and 0.1 mg/mL bacitracin (Serva).

*Buffer II* (used for binding experiments at the Y_1_R, Y_2_R, and Y_5_R): an isotonic sodium-containing HEPES buffer (10 mM HEPES, 150 mM NaCl, 25 mM NaHCO_3_, 2.5 mM CaCl_2_, 5 mM KCl, pH 7.4) supplemented with 1% BSA and 0.1 mg/mL bacitracin (Serva).

*DPBS* (used for binding experiments at Y_4_R): Dulbecco’s phosphate-buffered saline with calcium and magnesium (1.8 mM CaCl_2_, 2.68 mM KCl, 1.47 mM KH_2_PO_4_, 3.98 mM MgSO_4_, 137 mM NaCl, 8.06 mM Na_2_HPO_4_, pH 7.4) supplemented with 1% BSA and 0.1 mg/mL bacitracin.

Buffer for the Fura-2 assay: HEPES buffer (120 mM NaCl, 5 mM KCl, 2 mM MgCl_2_, 1.5 mM CaCl_2_, 25 mM HEPES and 10 mM glucose, pH 7.4) supplemented with 2% BSA and 2.5 mM probenecid.

## Radioligand Binding Assays

Radioligand competition binding assays, used to study Y_1_R, Y_2_R, Y_4_R, and Y_5_R binding of **2** and hPYY, were performed at intact YR expressing cells at 23 ± 2 °C. The radiochemical competition binding assays were recently validated by the determination of binding affinities of the reference ligands pNPY (Y_1_R, Y_2_R, and Y_5_R) and hPP (Y_4_R), which were in good agreement with previously reported binding affinities [for determined and reference p*K*_i_ or *K*_i_ values of pNPY (Y_1_R, Y_2_R, and Y_5_R), see Konieczny et al.;^43^ for determined and reference p*K*_i_ values of hPP (hY_4_R), see Gleixner et al.^33^].

### Y_1_R Binding

Competition binding assays at Y_1_R-expressing SK-N-MC neuroblastoma cells were performed as previously described using [^3^H]UR-MK299 as radioligand, but the used concentration of the radioligand was 0.075 nM instead of 0.15 nM.^47^ Prior to the competition binding experiments, the *K*_d_ value of [^3^H]UR-MK299 was determined by saturation binding at SK-N-MC cells according to the reported protocol (data not shown).^47^ The obtained *K*_d_ value amounted to 0.058 ± 0.007 nM (mean value ± SEM from five independent determinations performed in triplicate), being in good agreement with the originally reported dissociation constant (*K*_d_ = 0.044 nM).^47^ Total binding data (dpm, including total binding in the absence of competitor) were plotted against log(concentration competitor) and analyzed by a four-parameter logistic equation [log(inhibitor) vs response-variable slope, GraphPad Prism 5, GraphPad Software, San Diego, CA, USA], followed by normalization (100% = “top” of the four-parameter logistic fit, 0% = unspecifically bound radioligand) and analysis of the normalized data by a four-parameter logistic equation. pIC_50_ and IC_50_ values from individual experiments were converted to p*K*_i_ and *K*_i_ values according to the Cheng-Prusoff equation^46^ (logarithmic form in the case of p*K*_i_ values).

### Y_2_R Binding

Competition binding assays at CHO-hY_2_R cells were performed as previously described using [Lys^4^-[^3^H]propionyl]pNPY (*K*_d_ = 0.14 nM, concentration: 0.5 nM) as radioligand.^43^ Data were processed as described for the Y_1_R competition binding assay.

### Y_4_R Binding

Competition binding experiments at CHO-hY_4_R-G_qi5_-mtAEQ cells using [^3^H]UR-JG102 as radioligand (*K*_d_ = 0.11 nM, concentration: 0.25 nM) were performed as previously described.^33^ Data were processed as described for the Y_1_R competition binding assay.

### Y_5_R Binding

Competition binding studies at HEC-1B-hY_5_R cells using [Lys^4^-[^3^H]propionyl]pNPY (*K*_d_ = 11 nM,^28^ concentration: 5 nM) as radioligand were performed as previously reported.^53^ Data were processed as described for the Y_1_R competition binding assay.

## Y_2_R Binding Assays with [^3^H]**2**

Y_2_R binding studies with [^3^H]**2** were performed at intact CHO-hY_2_R-cells at 24 ± 2 °C. All experiments were performed in triplicate. Cell suspensions were prepared as follows: cells were grown in T75 flasks to reach a confluency of 80–90% on the day of the experiment. The culture medium was removed, and the adherent cells were washed twice with *buffer I* or *buffer II* (in this case, both buffers are not supplemented with BSA and bacitracin). For saturation binding and kinetic experiments, the cells were detached by trypsinization, suspended in *buffer I* or *buffer II* (without both BSA and bacitracin), and centrifuged at 300 g at rt for 5 min. For competition binding experiments, cells were scraped off the flask using a cell scraper and were centrifuged at 300 g at rt for 5 min. In both cases (trypsinization and scraping), the supernatant was discarded, and the cells were resuspended in *buffer I* or *buffer II* at varying densities (80,000–500,000 cells/mL) depending on the type of the experiment (for saturation binding experiments a low density was used to reduce the extent of ligand depletion). For all binding experiments, the same filtration procedure for separating free radioligand from cell-bound radioligand and for measuring the activity of the latter was used: after completed incubation, cells were collected on GF/C filter mats (0.26 mm; Whatman, Maidstone, UK) (pretreated with 0.3% polyethylenimine for 30 min) using an in-house manufactured harvester for 96 well plates (precision engineering workshop of the University of Regensburg, Regensburg, Germany), and the wells of the plate and the cells on the filter mat were immediately washed twice with ice-cold PBS. Filter pieces for each well were punched out and transferred into 1450–401 96-well sample plates (PerkinElmer, Rodgau, Germany), followed by the addition of Rotiscint Routine (Carl Roth, Karlsruhe, Germany) (200 μL). The plates were sealed with a transparent sealing tape (Greiner Bio-One EASYseal, part no. 676001; Greiner Bio-One, Frickenhausen, Germany) and shaken in the dark for at least 3 h before measurement. Radioactivity (dpm) was measured with a MicroBeta2 plate counter equipped with six pairs of photomultiplier tubes (PerkinElmer, Rodgau, Germany).

### Saturation Binding Experiments

The wells of a polypropylene 96-well plate (Brand, Wertheim, Germany) were prefilled with freshly prepared cell suspension (160 μL), followed by the addition of 20 μL of *buffer I* or *buffer II* (determination of total binding) or 20 μL of a solution of BIIE0246 and JNJ31020028 (10 μM each) in *buffer I* or *buffer II* (final concentrations: 1 μM each) (determination of unspecific binding) and the addition of 20 μL of a 10-fold concentrated (compared to the final concentration) solution of [^3^H]**2** in *buffer I* or *buffer II*. Samples were incubated under shaking for 2 or 4.5 h, followed by cell harvesting and further processing as described afore. Specific binding data, obtained by subtracting triplicate dpm mean values of unspecific binding from triplicate dpm mean values of total binding, were plotted against the free radioligand concentration and analyzed by a two-parameter equation describing hyperbolic single site binding (one-site, specific binding, GraphPad Prism 5) to obtain *K*_d_ values. The free concentration of [^3^H]**2** (nM) was calculated by subtracting the amount of specifically bound [^3^H]**2** (nM) (calculated from specifically bound [^3^H]**2** in dpm, the molar activity and the volume per well) from the total concentration of [^3^H]**2**.

### Association Experiments

The wells of a polypropylene round-bottom 96-well plate (Brand) were prefilled with freshly prepared cell suspension (160 μL). 20 μL of *buffer I* or *buffer II* (determination of total binding) or 20 μL of a solution of BIIE0246 and JNJ31020028 in *buffer I* (0.64 μM each) or *buffer II* (6.4 μM each) (final concentrations: 0.064 and 0.64 μM, respectively) (determination of unspecific binding) were added, followed by the addition of 20 μL of a 0.32 nM (*buffer I*) or 3.2 nM (*buffer II*) solution of [^3^H]**2** (final concentrations: 0.032 and 0.32 nM, respectively). Each association experiment was set up on a 96-well plate. Samples were incubated under shaking for different periods of time. The samples of the different time points were prepared in reversed order (longest incubation time first, shortest incubation time last) so that the cells of all samples could be collected simultaneously with the harvester (method described above). The studied timespan was 1–240 min for *buffer I* and 1–120 min for *buffer II*. Specific binding data, obtained by subtracting triplicate dpm mean values of unspecific binding from triplicate dpm mean values of total binding, were plotted against the time. In the case of *buffer I*, the association of [^3^H]**2** was clearly biphasic, reaching a first, well pronounced plateau at approximately 35 min, followed by a second association phase appearing at ca. 100 min (cf. Figure 4B). As the specific binding data of [^3^H]**2** could not be analyzed (ambiguous results) using the two-phase association fit (two-phase association, Y_0_ constrained to zero, GraphPad Prism 5), data were separately analyzed for the time intervals 1–100 min (initial fast association phase) and 100–240 min (second slow association phase). The data set 1–100 min was analyzed by a three-parameter equation describing an exponential rise to a maximum (one-phase association, Y_0_ constrained to zero, GraphPad Prism 5) to yield the observed association rate constant *k*_obs(bi,fast)_. The data set 100–240 min was analyzed by a four-parameter equation describing an exponential rise to a maximum starting from a plateau (plateau followed by one-phase association, X_0_ constrained to 100 min, Y_0_ constrained to the plateau value obtained by fitting of the 1–100 min data set, GraphPad Prism 5) to yield the observed association rate constant *k*_obs(bi,slow)_. To calculate mean values in % (cf. Figure 4B), specific binding data were normalized based on the B_eq_ (plateau) value (set to 100%) obtained by fitting the 100–240 min data set. Fitting of the mean values of the normalized data in the same manner as the analysis of the individual data sets (see above) gave the proportion of the fast association phase (43%, B_eq_ of the one-phase association fit). The difference to 100% corresponded to the proportion of the slow association phase (57%). Specific binding data of the association experiments performed in *buffer II*, neither indicating a clear monophasic or biphasic binding behavior, were analyzed by a five-parameter equation (two-phase association, Y_0_ constrained to zero, GraphPad Prism 5) to yield the observed association rate constants *k*_obs_ (note: the extra sum-of squares *F*-test (GraphPad Prism 5) supported the biphasic model over the monophasic fit, *P* < 0.0001). To calculate mean values in %, specific binding data were normalized based on the corresponding B_eq_ (plateau) value. Analysis of the mean values of the normalized data by a five-parameter equation (two-phase association, Y_0_ constrained to zero, GraphPad Prism 5) provided the proportion of the fast association phase (60%, “percentfast” GraphPad Prism 5). The difference to 100% corresponded to the proportion of the slow association phase (40%). In addition to nonlinear data fitting, data were fitted linearly by plotting ln[B_eq_/(B_eq_ – B_t_)] against the time (cf. Figure 4B).

### Dissociation Experiments

The wells of a polypropylene round-bottom 96-well plate (Brand) were prefilled with freshly prepared cell suspension (160 μL). 20 μL of *buffer I* or *buffer II* (determination of total binding) or 20 μL of a solution of BIIE0246 and JNJ31020028 in *buffer I* (3.2 μM each) or *buffer II* (8 μM each) (final concentrations: 320 and 800 nM, respectively) (determination of unspecific binding) were added, followed by the addition of 20 μL of a 1.6 nM (*buffer I*) or 4 nM (*buffer II*) solution of [^3^H]**2** (final concentrations: 0.16 and 0.4 nM, respectively) to start the preincubation. Each dissociation experiment was set up on a 96-well plate. Samples were incubated under shaking for 90 min in both buffers, followed by the addition of 20 μL of a solution of BIIE0246, JNJ31020028, and hNPY in *buffer I* (0.8 μM each) or *buffer II* (20 μM each) (final concentrations: 800 and 2000 nM, respectively) to initiate the dissociation. The incubation was continued under shaking. The samples of the different time points were prepared in reversed order (preincubation with the radioligand was precisely started 90 min before the start of the dissociation) so that the cells of all samples could be collected simultaneously with the harvester (method described above). The studied dissociation periods were 1–360 min for both buffers. Specific binding data, obtained by subtracting triplicate dpm mean values of unspecific binding from triplicate dpm mean values of total binding, were plotted against the time and analyzed by a three-parameter equation, describing a potentially incomplete monophasic exponential decline (one phase decay, GraphPad Prism 5) to obtain *k*_off_ and plateau values. The mean ± SEM of the plateau values from individual experiments proved to be throughout significantly different from zero (one-tailed *t*-test, *P* < 0.05). Thus, reanalysis of the data using a two-parameter equation (complete monophasic exponential decline) was not performed. To calculate mean values in % (cf. Figure 4C), binding data were normalized to Y_0_ [specifically bound ligand (*B*) at *t* = 0 (*B*_0_)]. In addition to nonlinear data fitting, data were fitted linearly by plotting ln[(*B*_t_ – *B*_plataeu_)/(*B*_0_ – *B*_plateau_)] against the time (cf. Figure 4C).

### Calculation of Association Rate Constants (*k*_on_) and *K*_d_(kin)

The association rate constants were calculated according to the equation *k*_on_ = (*k*_obs_ – *k*_off_)/[radioligand], where [radioligand] represents the concentration of [^3^H]**2** used for association experiments and *k*_obs_ and *k*_off_ the mean values of the individually determined *k*_obs_ and *k*_off_ values. The kinetically derived dissociation constants *K*_d_(kin) of [^3^H]**2** were calculated according to *K*_d_(kin) = *k*_off_/*k*_on_, where *k*_on_ and *k*_off_ represent the *k*_on_ and *k*_off_ mean values calculated from individual *k*_on_ and *k*_off_ values.

### Competition Binding Experiments

The wells of a polypropylene round-bottom 96-well plate (Brand) were prefilled with freshly prepared cell suspension (160 μL). 20 μL of a 10-fold concentrated (relative to the final concentration) solution of the compound of interest in *buffer I* or *buffer II* and 20 μL of a 10-fold concentrated solution of [^3^H]**2** in *buffer I* or *buffer II* were added (used concentrations of [^3^H]**2**: 0.05 nM in *buffer I* and 0.4 nM in *buffer II*). To determine total binding in the absence of a competitor, 20 μL of *buffer I* or *buffer II* were added, followed by the addition of 20 μL of the aforementioned radioligand solution. To determine unspecific binding, 20 μL of a solution of BIIE0246 and JNJ31020028 in *buffer I* or *buffer II* (in *buffer I*: 1 μM each; in *buffer II*: 8 μM each; final concentrations: 100 and 800 nM, respectively) and 20 μL of the aforementioned radioligand solution were added. Samples were incubated under shaking for 2 h, followed by cell harvesting and further processing as described afore. Total binding data (dpm, including total binding in the absence of competitor) were plotted against log(concentration competitor) and analyzed by a four-parameter logistic equation [log(inhibitor) vs response-variable slope, GraphPad Prism 5], followed by normalization (100% = “top” of the four-parameter logistic fit, 0% = unspecifically bound radioligand) and analysis of the normalized data by a four-parameter logistic equation. pIC_50_ and IC_50_ values were converted to p*K*_i_ and *K*_i_ values according to the Cheng–Prusoff equation^46^ (logarithmic form in the case of p*K*_i_ values) using the *K*_d_ values of 0.016 nM (*buffer I*) and 0.16 nM (*buffer II*).

## Fura-2 Ca^2+^ Assay

The Fura-2 Ca^2+^ assay was performed with CHO-hY_2_R cells using a protocol reported for a neurotensin receptor 1 Fura-2 Ca^2+^ assay (CHO-hNTS_1_R cells)^56^ with minor modifications: black 96-well plates (Greiner Bio-One 655076, Kremsmünster, Austria) were used instead of white 96-well plates, measurements (top read) in the plate reader (CLARIOstar Plus Microplatereader; BMG Labtech, Ortenberg, Germany) were carried out at 27 °C instead of 37 °C, and a measurement of one well comprised 28 cycles (47 s) instead of 44 cycles. Data were processed as reported.^56^ For the normalization, the response elicited by 30 μM hPYY was set to 100% (maximal response).

### Statistical Significance

For the applied statistical tests (*F*-test and *t*-test), the significance level was set to *P* ≤ 0.05.

## Calculation of Propagated Errors

Propagated errors [applying to specifically bound radioligand (saturation binding), association rate constants *k*_on_, and kinetically derived dissociation constants *K*_d_(kin)] were calculated as described elsewhere.^33^

## Supporting Information Available

Synthesis of [Lys^4^-(2-fluoropropionyl)]hPYY (Scheme S1); Structures of Y_2_R in complex with hNPY and hPYY(3–36) (Figure S1); RP-HPLC chromatogram of crude **2** (Figure S2); ^19^F NMR spectrum of [Lys^4^-(2-fluoropropionyl)]hPYY (Figure S3); ^19^F NMR spectrum of a mixture of **3** and TFA (Figure S4); chromatograms of the investigation of the chemical stability of **2** in PBS (Figure S5); RP-HPLC chromatogram of the micropreparative HPLC workup of [^3^H]**2** (Figure S6); radioligand displacement curves from competition binding experiments with hPYY and **2** at Y_1_R, Y_2_R, Y_4_R, and Y_5_R using reported radioligands (Figure S7); data of a representative saturation binding experiment with [Lys^4^-[^3^H]propionyl]pNPY at CHO–Y_2_R cells in sodium-containing buffer (Figure S8); concentration-response curves of hPYY and **2** obtained from a Y_2_R Fura-2 Ca^2+^ assay (Figure S9); and different fits of the data from Y_2_R association experiments in *buffer I* (Figure S10) (PDF)

## Abbreviations USED

BSA: bovine serum albumin
CHO: Chinese hamster ovary
DIPEA: *N*,*N*-diisopropylethylamine
FBS: fetal bovine serum
HEC: human endometrial cancer
HEPES: 4-(2-hydroxyethyl)-1-piperazineethanesulfonic acid
hPP: human pancreatic polypeptide
hY_x_R: human NPY Y_x_ receptor
*k*: retention (or capacity) factor (HPLC)
PBS: phosphate-buffered saline
p*K*_i_: negative decadic logarithm of the dissociation constant *K*_i_ (in M) obtained from a competition binding experiment
hNPY: human neuropeptide Y
pNPY: porcine neuropeptide Y
hPYY: human peptide YY
pPYY: porcine peptide YY
RP: reversed phase
SEM: standard error of the mean
*t*_R_: retention time
YR: NPY receptor.
