# Supplementary material for: Characterization of [3H]Propionylated Human Peptide YY-A New Probe for Neuropeptide Y Y2 Receptor Binding Studies
Source: ACS Pharmacol Transl Sci. 2025 Feb 25;8(3):785–99. doi: 10.1021/acsptsci.4c00666 (PMC11915035; doi:10.1021/acsptsci.4c00666)
Supplement: Supplementary file 1 — pt4c00666_si_001.pdf [file pt4c00666_si_001.pdf]

Supporting Information

Characterization of [^{3}H]Propionylated Human Peptide YY – A New Probe for Neuropeptide Y Y_2 Receptor Binding Studies

Franziska Schettler,[a] *Albert O. Gattor,*[a] *Pierre Koch*[a] *and Max Keller**[,a]

[a]Institute of Pharmacy, Faculty of Chemistry and Pharmacy, University of Regensburg, Universitätsstraße 31, D-93053, Germany

*E-mail address:

max.keller@chemie.uni-regensburg.de

Content Page

Scheme S1	S-2
Figures S1-S10	S-2
References	S-7

Scheme S1. Synthesis of [Lys^4-(2-fluoropropionyl)]hPYY (**4**).

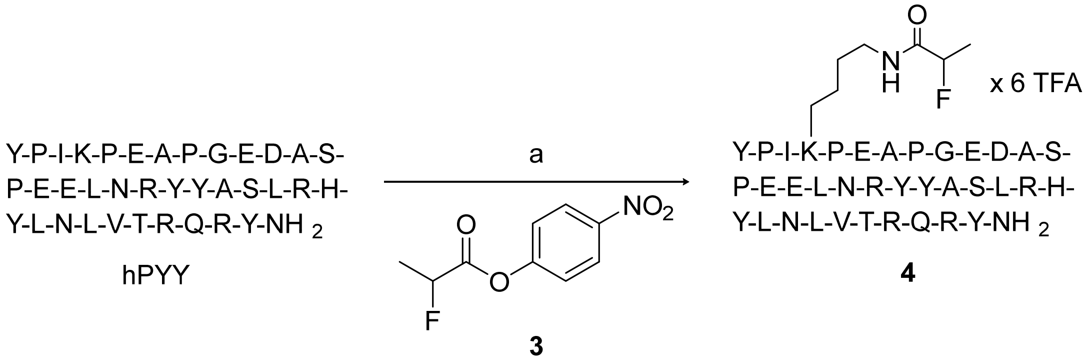

Reagents and conditions: (a) DIPEA, DMF/H_2O 80:20, rt, 1.5 h, 18%.

Figures S1-S10

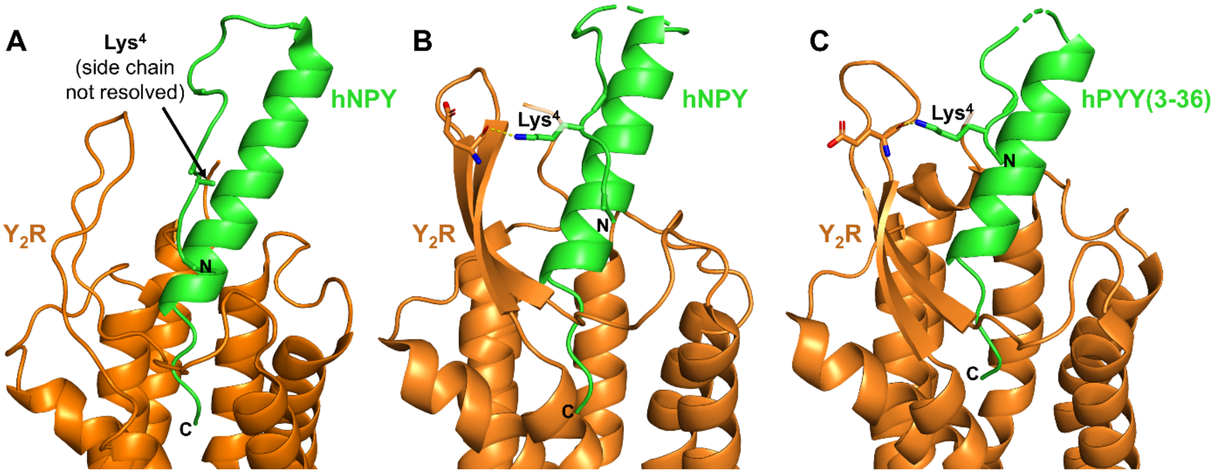

Figure S1. Structures of the Y_2R in complex with hNPY (A, B) or hPYY(3-36) (C) reproduced from the PDB IDs 7X9B[1] (A), 8K6N[2] (B) and 7YON[3] (C). Whereas in A no interaction of Lys^4 with the Y_2R was observed, an interaction of Lys^4 of NPY and PYY with the backbone NH of Glu193 of the Y_2R was observed in the Cryo-EM structures B and C, respectively. However, an interaction of the N-terminal section of NPY and PYY with the Y_2R is known to be of minor importance for Y_2R binding.[4, 5] Consequently, propionylation of the side chain of Lys^4 should not affect Y_2R binding of PYY.

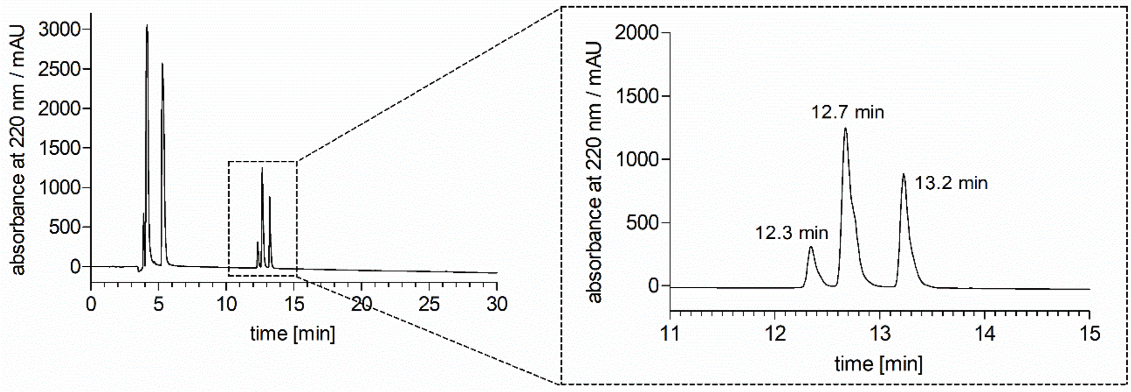

Figure S2. Chromatogram of the RP-HPLC analysis of crude **2** obtained by the treatment of 1 equiv. of hPYY with 1.5 equiv. of succinimidyl propionate (synthesis of **2**), showing a separation of the precursor hPYY (t_R = 12.3 min), **2** (t_R = 12.7 min) and the side product twofold propionylated hPYY (t_R = 13.2 min). The identity of the peptides was confirmed by LC-HRMS analysis (data not shown). The system and the conditions for the HPLC analysis are provided under *Analytical HPLC* (main article, experimental section).

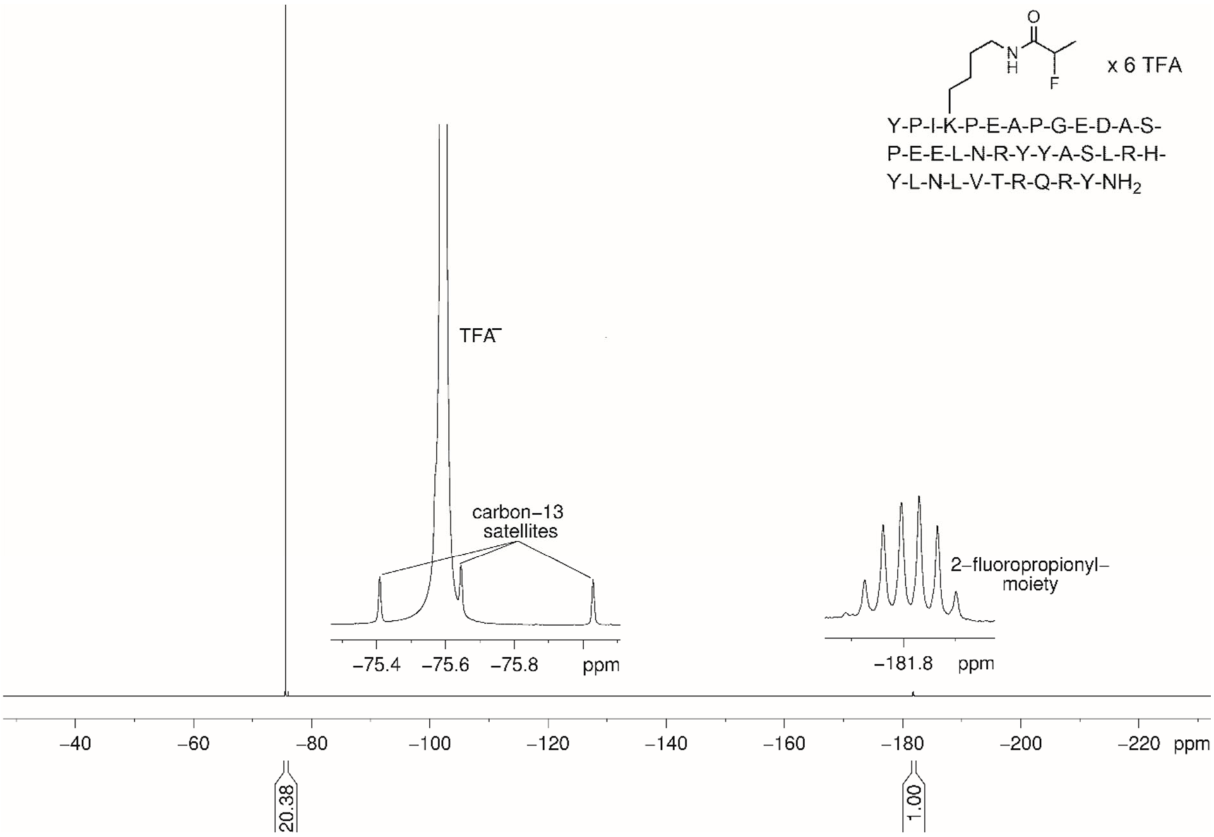

Figure S3. ^{19}F NMR spectrum of [Lys^{4}-(2-fluoropropionyl)]hPYY hexakis(hydrotrifluoroacetate) (**4**) in D_2O recorded on a Bruker Avance Neo 500 MHz spectrometer (^{19}F: 470 MHz, probe: CPP1.1 BBO 500S1 BB-H&F-D-05 Z XT; Bruker, Karlsruhe, Germany) at 298 K. ^{19}F chemical shifts were referenced based on the deuterium lock (D_2O).

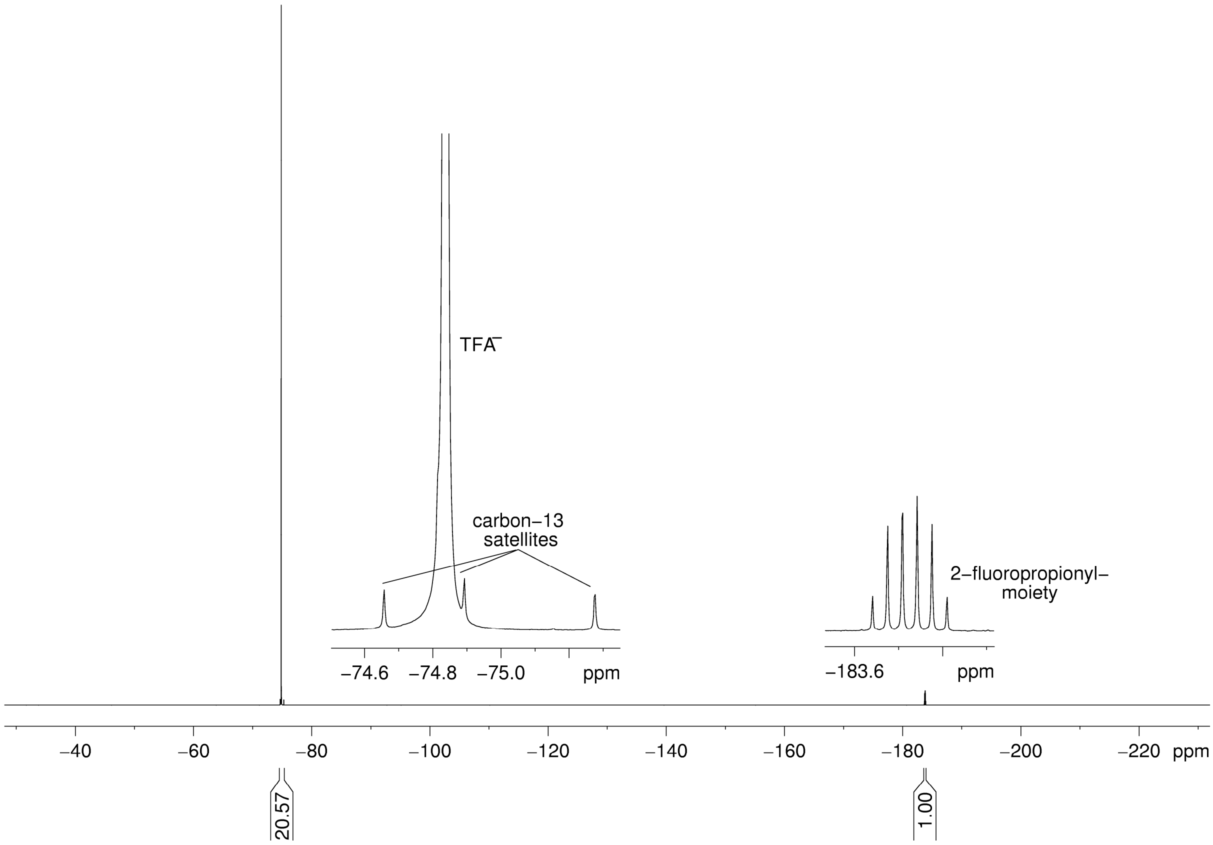

Figure S4. ^{19}F NMR spectrum of a solution of **3** (0.39 µmol) and TFA (2.34 µmol) (molar ratio 1:6) in D_2O recorded on a Bruker Avance Neo 500 MHz spectrometer (^{19}F: 470 MHz, probe: CPP1.1 BBO 500S1 BB-H&F-D-05 Z XT; Bruker, Karlsruhe, Germany) at 298 K. ^{19}F chemical shifts were referenced based on the deuterium lock (D_2O).

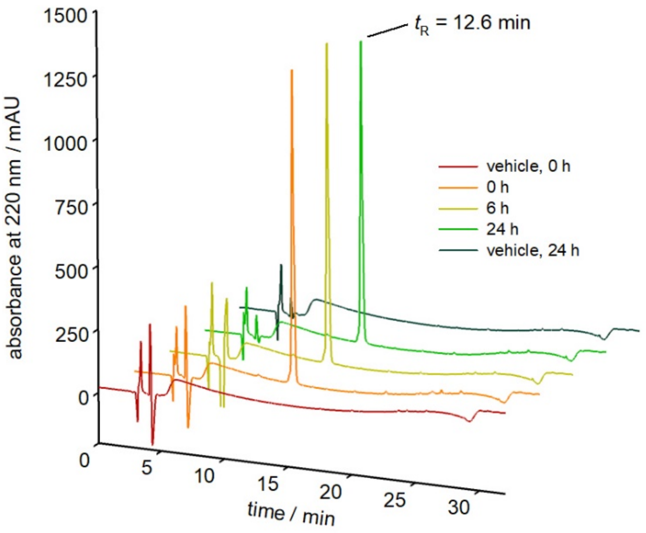

Figure S5. Chromatograms of the RP-HPLC analysis of **2** after incubation in PBS pH 7.4 at 24 °C for up to 24 h. **2** showed no decomposition.

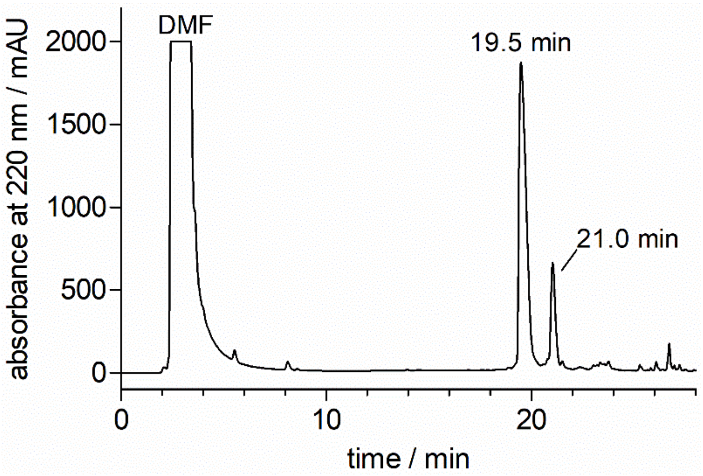

Figure S6. Chromatogram of a micropreparative RP-HPLC run performed to isolate [^{3}H]**2** from the mixture obtained by treatment of an excess of hPYY (3.2 equiv.) with succinimidyl [^{3}H]propionate. The radioligand [^{3}H]**2** (t_R = 21.0 min) could be separated from the labeling precursor hPYY (t_R = 19.5 min). The identity of purified [^{3}H]**2** was confirmed by RP-HPLC analysis of a mixture of [^{3}H]**2** and **2** (see Figure 3A, main article). The HPLC system and conditions are provided under *Synthesis of [^{3}H]**2*** (main article, experimental section).

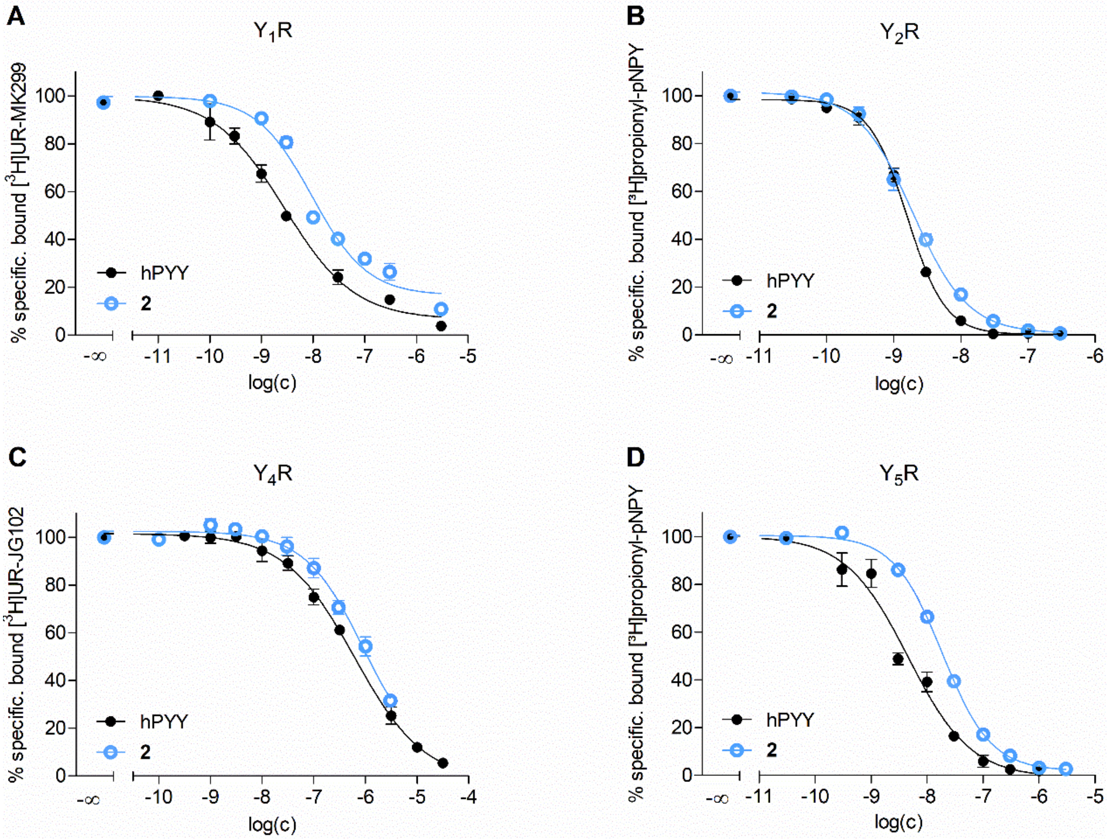

Figure S7. Radioligand displacement curves from competition binding experiments performed with hPYY and **2** at Y_1, Y_2, Y_4 or Y_5 receptor expressing cells. (A) Y_1 receptor expressing SK-N-MC neuroblastoma cells; radioligand: [^{3}H]UR-MK299 (K_d = 0.058 nM, c = 0.075 nM). (B) CHO-hY_2R cells; radioligand: [Lys^4-[^{3}H]propionyl]pNPY (K_d = 0.14 nM,[6] c = 0.5 nM). (C) CHO-hY_4R-Gq$_{i5}$-mtAEQ cells; radioligand: [^{3}H]UR-JG102 (K_d = 0.11 nM,[7] c = 0.25 nM). (D) HEC-1B-hY_5R cells; radioligand: [Lys^4-[^{3}H]propionyl]pNPY (K_d = 11 nM,[8] c = 5 nM). Data represent mean values ± SEM from three to five independent experiments (performed in triplicate).

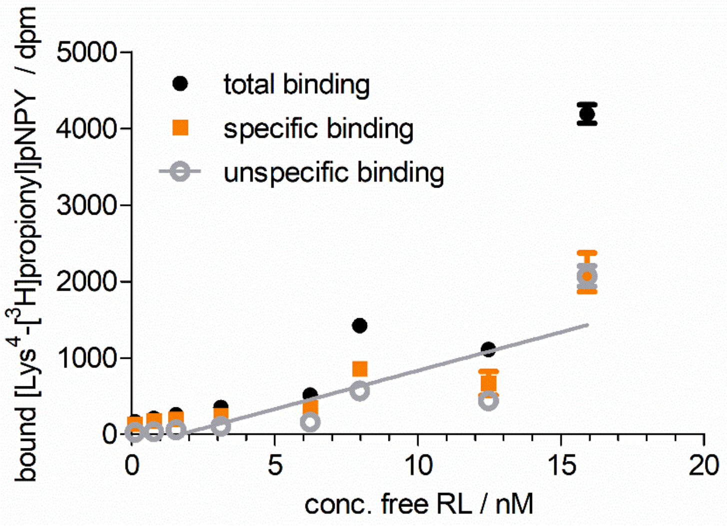

Figure S8. Data of a representative saturation binding experiment performed with [Lys^{4}-[^{3}H]propionyl]pNPY at CHO-Y_2R cells in sodium-containing buffer (DPBS, 145 mM Na^{+}). Total and unspecific binding data represent mean values ± SEM. Specific binding data represent calculated values ± propagated error. Specific binding data could not be fitted according to an equation describing a hyperbolic binding curve (no saturable binding). Unspecific binding data were fitted by linear regression.

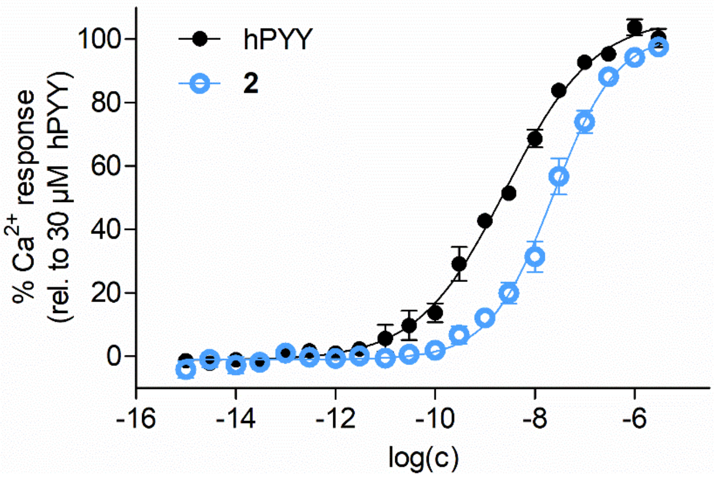

Figure S9. Concentration-response curves of hPYY and **2**, obtained from a Fura-2 Ca^{2+} assay performed with CHO-hY_2R cells. Mean values ± SEM from three independent experiments (performed in triplicate). The pEC_{50} values ± SEM amounted to 8.45 ± 0.05 (hPYY) and 7.56 ± 0.09 (**2**).

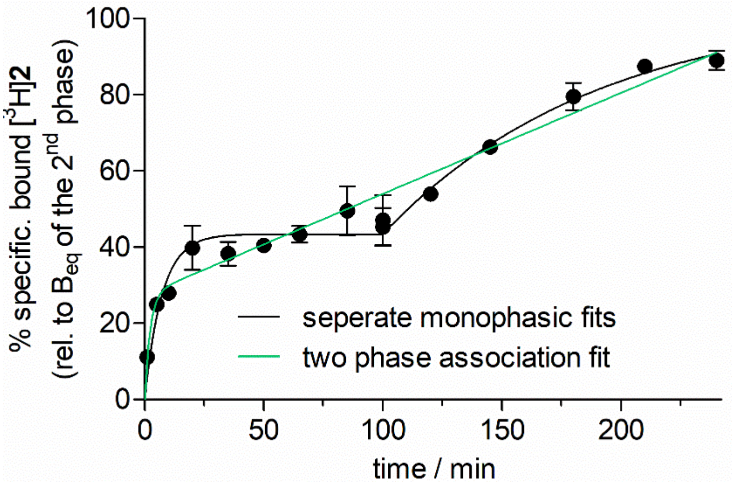

Figure S10. Comparison of different fits (separate monophasic exponential fits for the slow and fast association phase vs. two phase association fit, GraphPad Prism 5) of the data obtained from Y_2R association experiments with [^{3}H]**2** performed in sodium-free *buffer I.*

References

1. Tang, T.; Tan, Q.; Han, S.; Diemar, A.; Lobner, K.; Wang, H.; Schuss, C.; Behr, V.; Morl, K.; Wang, M.; Chu, X.; Yi, C.; Keller, M.; Kofoed, J.; Reedtz-Runge, S.; Kaiser, A.; Beck-Sickinger, A. G.; Zhao, Q.; Wu, B. Receptor-Specific Recognition of NPY Peptides Revealed by Structures of NPY Receptors. *Sci. Adv.* **2022**, *8*, eabm1232.
2. Shen, S.; Deng, Y.; Shen, C.; Chen, H.; Cheng, L.; Wu, C.; Zhao, C.; Yang, Z.; Hou, H.; Wang, K.; Shao, Z.; Deng, C.; Ye, F.; Yan, W. Structural Basis of Neuropeptide Y Signaling Through Y_1 and Y_2 Receptors. *MedComm* **2024**, *5*, e565.
3. Kang, H.; Park, C.; Choi, Y. K.; Bae, J.; Kwon, S.; Kim, J.; Choi, C.; Seok, C.; Im, W.; Choi, H. J. Structural Basis for Y_2 Receptor-Mediated Neuropeptide Y and Peptide YY Signaling. *Structure* **2023**, *31*, 44-57.e6.
4. Cabrele, C.; Beck-Sickinger, A. G. Molecular Characterization of the Ligand-Receptor Interaction of the Neuropeptide Y Family. *J. Pept. Sci.* **2000**, *6*, 97-122.
5. Xu, B.; Vasile, S.; Ostergaard, S.; Paulsson, J. F.; Pruner, J.; Aqvist, J.; Wulff, B. S.; Gutierrez-de-Teran, H.; Larhammar, D. Elucidation of the Binding Mode of the Carboxyterminal Region of Peptide YY to the Human Y_2 Receptor. *Mol. Pharmacol.* **2018**, *93*, 323-334.
6. Konieczny, A.; Braun, D.; Wifling, D.; Bernhardt, G.; Keller, M. Oligopeptides as Neuropeptide Y Y_4 Receptor Ligands: Identification of a High-Affinity Tetrapeptide Agonist and a Hexapeptide Antagonist. *J. Med. Chem.* **2020**, *63*, 8198-8215.

7. Gleixner, J.; Gattor, A. O.; Humphrys, L. J.; Brunner, T.; Keller, M. [^{3}H]UR-JG102-A Radiolabeled Cyclic Peptide with High Affinity and Excellent Selectivity for the Neuropeptide Y Y_4 Receptor. *J. Med. Chem.* **2023**, *66*, 13788-13808.
8. Dukorn, S.; Littmann, T.; Keller, M.; Kuhn, K.; Cabrele, C.; Baumeister, P.; Bernhardt, G.; Buschauer, A. Fluorescence- and Radiolabeling of [Lys^4,Nle^{17,30}]hPP Yields Molecular Tools for the NPY Y_4 Receptor. *Bioconjugate Chem.* **2017**, *28*, 1291-1304.
